# Double quick, double click reversible peptide “stapling”†
†Electronic supplementary information (ESI) available: Synthesis and characterization, additional biophysical and biochemical analyses. See DOI: 10.1039/c7sc01342f



**DOI:** 10.1039/c7sc01342f

**Published:** 2017-05-31

**Authors:** Claire M. Grison, George M. Burslem, Jennifer A. Miles, Ludwig K. A. Pilsl, David J. Yeo, Zeynab Imani, Stuart L. Warriner, Michael E. Webb, Andrew J. Wilson

**Affiliations:** a School of Chemistry , University of Leeds , Woodhouse Lane , Leeds LS2 9JT , UK . Email: A.J.Wilson@leeds.ac.uk; b Astbury Centre For Structural Molecular Biology , University of Leeds , Woodhouse Lane , Leeds LS2 9JT , UK

## Abstract

The development of constrained peptides for inhibition of protein–protein interactions is an emerging strategy in chemical biology and drug discovery. This manuscript introduces a versatile, rapid and reversible approach to constrain peptides in a bioactive helical conformation using BID and RNase S peptides as models. Dibromomaleimide is used to constrain BID and RNase S peptide sequence variants bearing cysteine (Cys) or homocysteine (*h*Cys) amino acids spaced at *i* and *i* + 4 positions by double substitution. The constraint can be readily removed by displacement of the maleimide using excess thiol. This new constraining methodology results in enhanced α-helical conformation (BID and RNase S peptide) as demonstrated by circular dichroism and molecular dynamics simulations, resistance to proteolysis (BID) as demonstrated by trypsin proteolysis experiments and retained or enhanced potency of inhibition for Bcl-2 family protein–protein interactions (BID), or greater capability to restore the hydrolytic activity of the RNAse S protein (RNase S peptide). Finally, use of a dibromomaleimide functionalized with an alkyne permits further divergent functionalization through alkyne–azide cycloaddition chemistry on the constrained peptide with fluorescein, oligoethylene glycol or biotin groups to facilitate biophysical and cellular analyses. Hence this methodology may extend the scope and accessibility of peptide stapling.

## Abstract 2

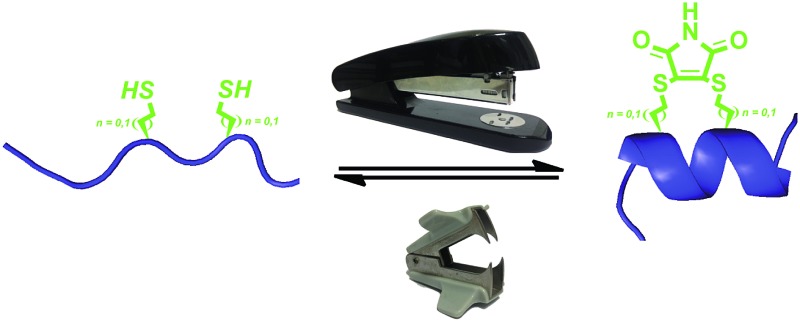
A versatile, rapid and reversible approach to constrain peptides in a bioactive helical conformation and bearing a functional handle for inhibition of protein–protein interactions is described.

## Introduction

**Fig. 1:**
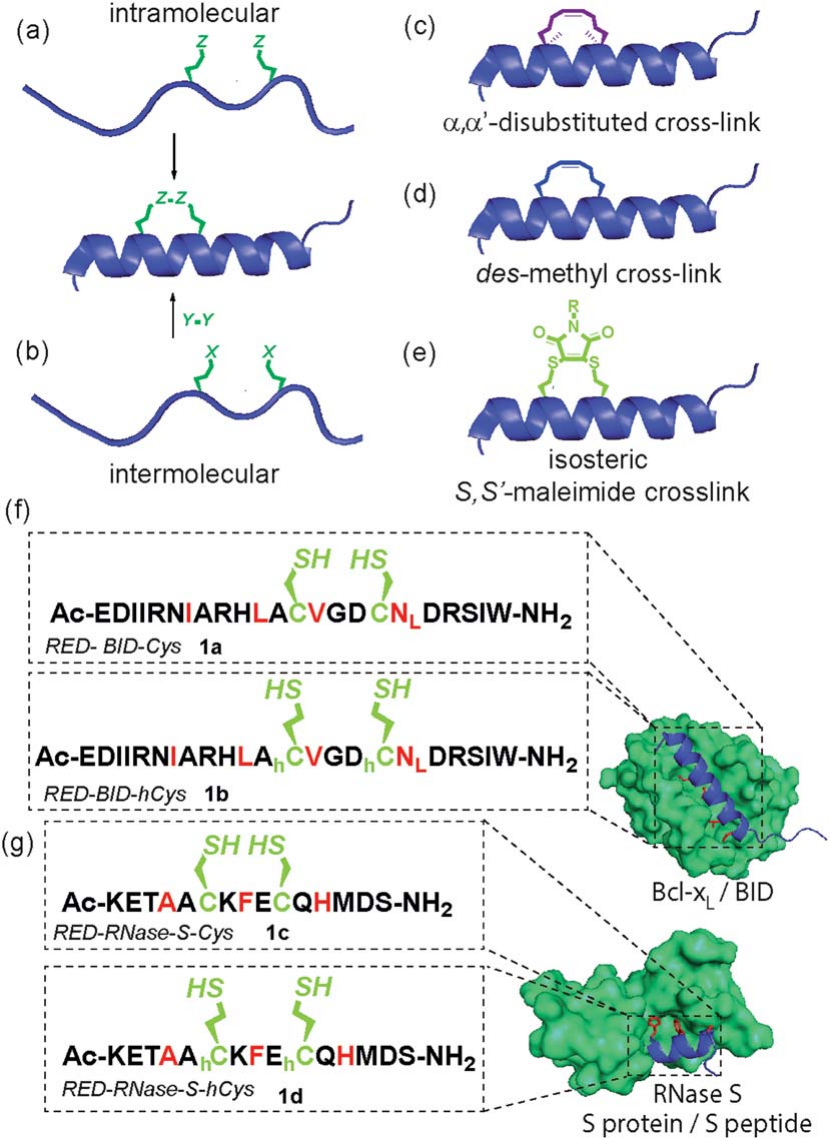
Overview of peptide stapling strategies and linkers for constraining peptides in an α-helical conformation together with structures for the model protein–protein interactions used in this study; (a) schematic for intramolecular crosslinking; (b) schematic for intermolecular crosslinking; (c) *S*,*S*-α,α′-disubstituted *i*, *i* + 4 hydrocarbon “staple”; (d) *S*,*S-des*-methyl *i*, *i* + 4 hydrocarbon constraint; (e) *i*, *i* + 4 *S*,*S*′-maleimide staple reported in this work; (f) and (g) model peptide sequences **1a–d** for BID (**1a–b**) and RNase S (**1c–d**) and protein–protein interactions in this study (Bcl-x_L_ (green)/BID (blue) PDB ID: ; 4QVE; RNase S protein (green)/S peptide (blue) PDB ID: ; 1CJQ; hotspot residues (red)).

Inhibition of α-helix mediated protein–protein interactions (PPIs)^1–4^ represents an area of intense interest, due to their role in intracellular signalling processes^5^ and demonstrated tractability for drug discovery.^6,7^ The last decade has seen an increasing emphasis on the development of constrained peptides^8–10^ for PPI inhibition. Constraint of a peptide in a bioactive conformation is proposed to improve target binding affinity (by preorganization),^11,12^ increase stability (*e.g.* by protecting against proteolysis)^13–15^ and enhance cell-uptake,^16,17^ although a number of recent studies have provided conflicting evidence for the enhanced cell permeability and cellular potency that is conferred through stapling.^18–20^ Available strategies^9,13,21–25^ to constrain a peptide in a helical conformation (Fig. 1a–e), include hydrocarbon “staples”,^26–28^ lactam bridges,^29–31^ hydrogen-bond surrogates,^32^ photoswitches^11,33,34^ and triazoles introduced by “click” chemistry.^35–38^ The nature of the linker, including any stereocenters, plays a role in regulating peptide conformation and target protein affinity.^31,39^ Introduction of constraints may be achieved either by intramolecular reaction (Fig. 1a)^28,31^ or by chemoselective intermolecular reaction (Fig. 1b) between suitably disposed residues within the peptide sequence and an appropriate coupling partner.^36,40^ Introduction of a constraint by intermolecular reaction, whilst synthetically more demanding, may be advantageous in that it provides access to functionalized constraints^36^ and thus to peptides tailored for biophysical, cellular and chemical proteomics analyses. Herein, we use BID and RNase S variant peptides (**1a–d**) which respectively bind Bcl-x_L_ proteins^41^ and RNase S protein^42^ (Fig. 1f and g), to demonstrate that dibromomaleimides^43–51^ serve as versatile constraints that can be introduced and removed.^52–57^ These reagents have not previously been studied for their ability to control peptide helicity. Introduction of an alkyne onto the dibromomaleimide permits further divergent functionalization by “click” chemistry.^58^ This approach may therefore complement recently reported methods to constrain peptides by reaction of dichloroacetone^59^ with side chain thiols and photo-reversible constraints using *S*,*S*′-tetrazine.^60^

Previously, we^14^ and others^61^ introduced α-pentenylglycine as an alternative to the widely used α,α′-pentenylalanine for cross-linking the *i* and *i* + 4 residues within a peptide (Fig. 1c and d).^26^ Production of this *des*-methyl constraint requires a more easily synthesized amino acid which is less sterically hindered for peptide coupling, and, as shown by us, has no deficiencies in terms of biophysical properties when applied to BH3 peptides BID and BIM.^62^ We hypothesized that this all hydrocarbon 8-atom constraint could be replaced with an isoatomic *S*,*S*′-maleimide crosslink by reaction of suitably disposed homocysteine (*h*Cys) residues with dibromomaleimide (or for cysteine (Cys), a 6-atom constraint) (Fig. 1e).

**Fig. 2:**
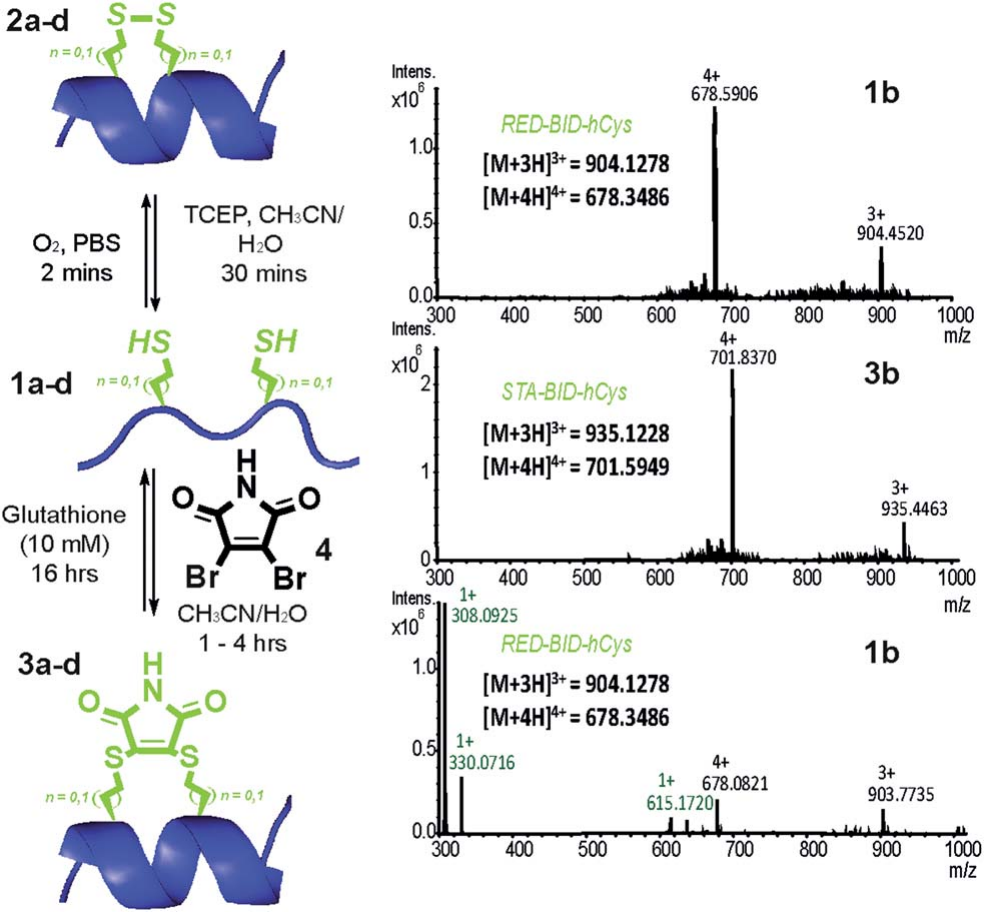
Reversible constraining reaction sequence for peptides **1–3a–d**: conditions for conversion from the oxidised state (OX) through the intermediate reduced state (RED) to the constrained peptide (STA) and *vice versa* (LHS); HRMS analyses of reaction mixture for reversible interconversion between **1b** and **3b** (relevant masses are indicated in black for the peptide and in green for excess glutathione used during unconstraining) (RHS).

Peptides **1a–d** bearing Cys or *h*Cys amino acids in positions used previously^26,62^ for *i* → *i* + 4 constraints were independently oxidised to the constrained disulfide **2a–d** or cross-linked with dibromomaleimide **4** to generate **3a–d** with rapid and complete conversion. Alternatively, this sequence **1a–d** to **3a–d** could be performed in one pot and peptides **1a–d** fully regenerated by addition of an excess of thiol (Fig. 2 and S4–7†). It is noteworthy that conversion of the Cys-derived sequences **1a** and **1c** to their constrained derivatives **3a** and **3c** was more rapid than for the corresponding *h*Cys derivatives **1b** and **1d**. It should be noted that these studies were carried out in unbuffered aqueous solution and future studies will focus on a more detailed analysis of the reaction rates, their pH dependence and comparison to other rapid constraining reactions.^63^ Of further note is the fact that a range of thiols were used including a 10 mM aqueous solution of glutathione, which corresponds to the average concentration present in cancer cells.^64,65^ It may thus be feasible to harness this approach for controlled delivery of a peptide to the diseased cell; a focus of future studies. Interestingly, removal of the maleimide cross-link could be abrogated through addition of an excess of the reducing agent TCEP, resulting in a permanent succinimide cross-link (Fig. S9†). Succinimide linkages of a similar nature have been shown to undergo thiol mediated cleavage;^48^ future efforts to “lock” the constraint may therefore be more appropriately served by hydrolysis of the imide in the maleimide crosslink.^66^

**Fig. 3:**
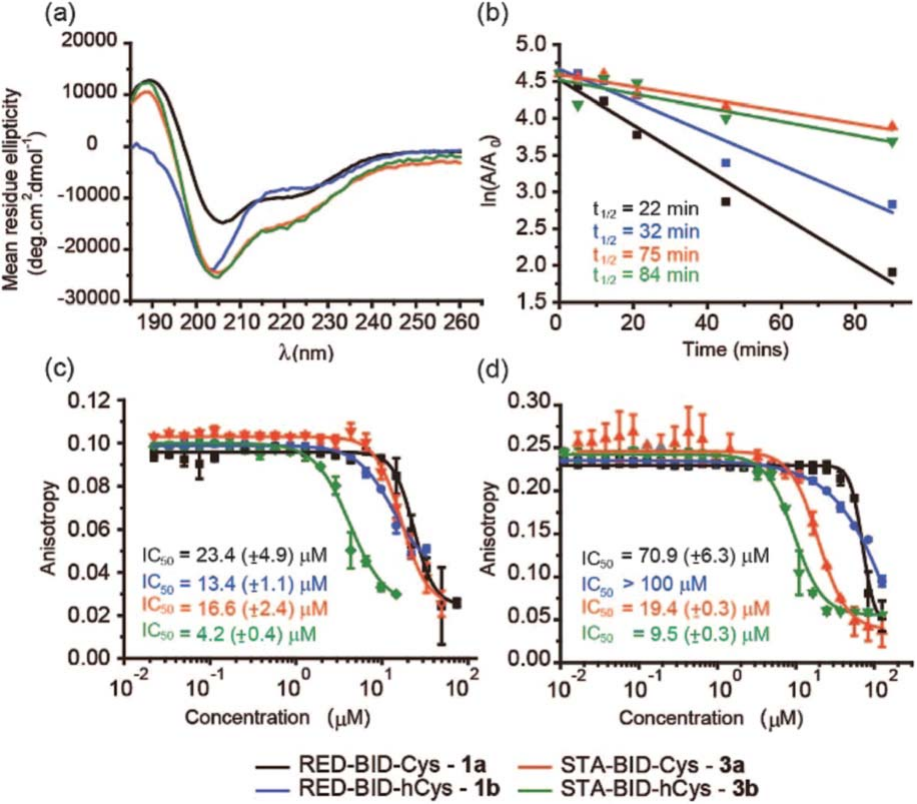
Analyses on BID derived peptides **1a,b** and **3a,b**. (a) Circular dichroism spectra (water/30% acetonitrile); (b) trypsin proteolysis in a 1 : 10 000 enzyme/substrate ratio (50 mM phosphate buffer, 200 mM NaCl, pH 7.50); (c) fluorescence anisotropy competition assay for inhibition of the FITC-BID/Mcl-1 interaction (25 nM tracer, 150 nM protein, 50 mM Tris buffer, 150 mM NaCl, 0.61% of Triton X-100, pH 7.40); (d) fluorescence anisotropy competition assay for inhibition of the BODIPY-BAK/Bcl-x_L_ interaction (25 nM tracer, 150 nM protein, 50 mM phosphate buffer, 200 mM NaCl, 0.02 mg mL^–1^ BSA, pH 7.50).

Peptides **1a,b** and **3a,b** were analysed by CD spectroscopy and subjected to proteolysis assays and fluorescence anisotropy competition assays for inhibition of the BID/Mcl-1, BAK/Bcl-x_L_ and NOXA-B/Mcl-1 interactions (Fig. 3, S19–23 and S29–30†). Constrained peptides **3a,b** showed no significant difference in helicity (42% and 43%) to each other as a result of the different cross-link length but both were more helical than the corresponding reduced peptide **1a** or **1b**, (24% and 29%). Molecular dynamics calculations were conducted on **1b** and **3b** – these further support the conclusion that the helical conformation predominates when constrained by the maleimide linker (Fig. S25 and S26†). Tryptic proteolysis as monitored by LC-MS^14^ established that the conformationally constrained peptides **3a,b** exhibit improved proteolytic resistance in comparison to the reduced peptides **1a,b**; proteolysis was retarded up to 7 residues removed from either side of the thiol residues (Fig. S19–23†). Taken together these data indicate that the maleimide constraint confers comparable enhancement of helicity and proteolytic resistance to the *des*-methyl hydrocarbon constraint reported by us earlier.^62^ Peptides **1a,b** and **3a,b** inhibited the BID/Mcl-1 interaction with comparable potency to the native **WT-BID 8** sequence (IC_50_ ∼ 15–25 μM) except for the constrained peptide **3b** which was slightly more potent (IC_50_ ∼ 4.2 μM), thus the constraining/unconstraining reagents have either a weak or no effect on the inhibitory potency for this PPI. Although inconsistent with the concept that preorganization should promote higher binding affinity, the result is consistent with our own recent studies on hydrocarbon-constrained peptides, which illustrated enthalpy-entropy compensation operates for these peptides which bind through an induced-fit mechanism.^62^ For inhibition of BAK/Bcl-x_L_ both constrained peptides **3a,b** were shown to be significantly superior inhibitors compared to the reduced peptides **1a,b**. The differential effects upon potency for the same peptide against Bcl-x_L_ and Mcl-1 may arise as a consequence of favourable non-covalent contacts between the maleimide and Bcl-x_L_; it is anticipated that future structural analyses will shed light on these differences.

**Fig. 4:**
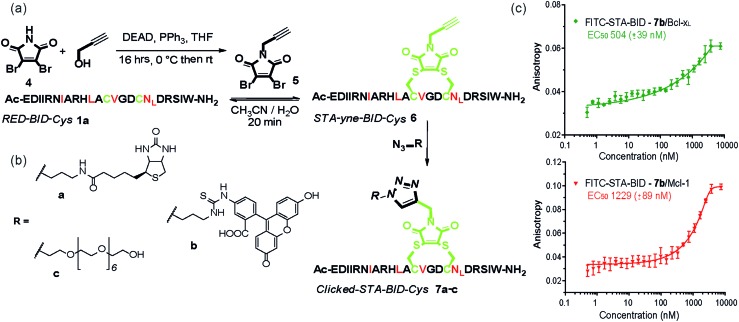
(a) Scheme illustrating double click chemistry for generating functionalized constrained peptides **7a–c**. An alkyne-maleimide was used to generate yne-STA-BID-Cys **6**, which was further functionalized by click reaction with (b) various azide derivatives. (c) Fluorescence anisotropy direct binding assay for interaction of FITC-STA-BID **7b** with Mcl-1 and Bcl-x_L_ (75 nM tracer, 15 μM protein, 50 mM Tris buffer, 150 mM NaCl, 0.61% of Triton X-100, pH 7.40).

The synthetic method was further adapted to allow the functionalization of the BID–Cys peptides **7a–c**. Using Mitsunobu alkylation,^67^ alkyne–dibromomaleimide **5** was prepared and conjugated to peptide **1a** to generate the constrained peptide **6**. Without isolation or purification, subsequent “click” reaction with biotin, fluorescein and PEG azides^38^ afforded complete conversion to click-conjugated constrained peptides **7a–c** (Fig. 4a and b). Related “clickable maleimides” have previously been used to conjugate small molecule drugs to polymers,^68^ whilst “clicked” dibromomaleimides have been used to conjugate polymers to oxytocin so as to improve solubility.^51^ Here we have expanded this methodology, demonstrating that this functionality can be used to constrain peptide conformation and as a divergent method for peptide functionalization. The utility of these modifications was demonstrated through a direct fluorescence anisotropy titration with the fluorescein linked peptide **7b** (Fig. 4c). The observed potencies are consistent with the EC_50_ values determined in the competition assays for **3a,b** indicating no detrimental consequence from the introduction of a fluorophore in this position. Such a modification could thus facilitate cellular studies whilst leaving the N-terminus of the peptide free to allow further orthogonal derivatization.

**Fig. 5:**
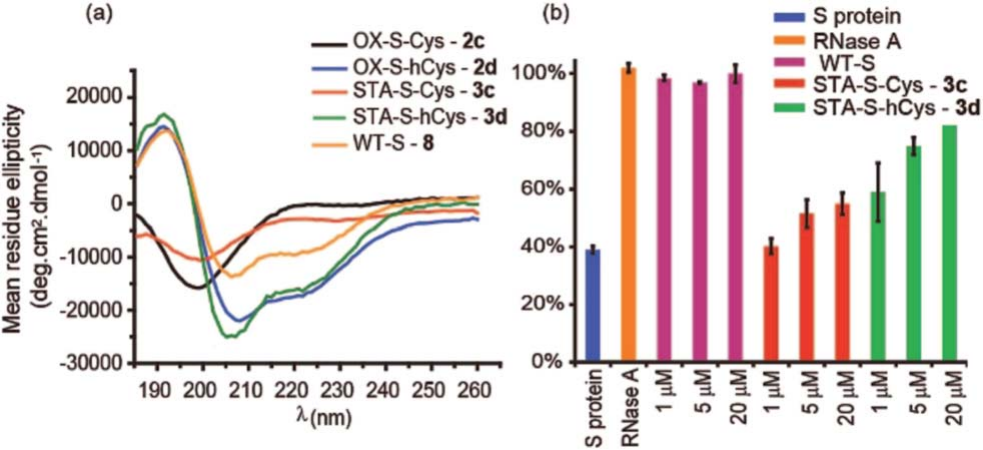
Biophysical and bioactivity analyses of WT-S and S derived peptides **3–4a,c**. (a) Circular dichroism spectra recorded in water (30% TFE); (b) RNA degradation assay showing the restoration of S protein activity. S protein was used as a negative control, and RNase A as a positive control. (50 mM Tris, 100 mM NaCl, pH 7.50).

The use of dibromomaleimide-based constraints for promotion of α-helicity was then further demonstrated using the RNase S-protein/S-peptide interaction with the choice of amino acid positions at which to introduce the constraint informed by previous studies by the Verdine,^26^ Hamachi^69^ and Dawson^59^ groups. S peptides **1c** and **1d** bearing Cys and *h*Cys residues at the *i* and *i* + 4 positions were synthesized using standard solid-phase synthesis in good yield and purified by HPLC (Fig. S2 and S3†). Peptides **1c** and **1d** could not readily be isolated as reduced bis-thiols; one-pot reduction followed by the reaction with dibromomaleimide **4** afforded peptides **3c** and **3d** (Fig. 2). Both peptides were then analysed by CD spectroscopy and used in an RNA degradation assay to determine the restoration of enzymatic activity to S-protein in place of the S-peptide (Fig. 5a and b). In this instance, different and more pronounced behaviour for the Cys and *h*Cys variants was observed, highlighting that the effects of introducing a constraint are context-dependent. The Cys-derived RNase S peptides **2–3c** exhibit poor helicity (*e.g.* **3c** = 9% *versus* wild-type **8** = 30%) and corresponding low restoration of enzymatic activity (Fig. S31†). Molecular modelling suggests that the introduction of a disulfide or a maleimide bridge does not support a helical conformation (Fig. S27 and S28†). In contrast, for the *h*Cys **1–3d** series introduction of a disulfide or a maleimide crosslink supports a helical structure comparable to the WT sequence and consistent with the moderate effects on helicity for an 8-carbon constraint in these positions. (Furthermore, and fully consistent with the work of Hamachi and co-workers,^69^ upon addition of trifluoroethanol (TFE), the constrained peptides reach a greater overall α-helical content). Here, introduction of a disulfide (**2d**) or a maleimide (**3d**) crosslink results in enhanced helicity in comparison to the WT sequence (**2d** = 56%, **3d** = 52% and wild-type **8** = 30%). As a result **3d** is much better able to restore RNase S protein activity than is **3c**.

## Conclusions

In conclusion, the attractiveness of thiol-based “stapling” approaches has been recognized for some time,^24^ but no general approach has been widely adopted; we have developed a new method to rapidly and reversibly introduce a functionalized constraint into two model peptides that enhances a range of biophysical and biochemical properties. Moreover, the constraint may be further functionalized in a divergent manner with motifs useful in chemical biology and proteomics applications. The approach can be carried out using natural readily available amino acid side chains on unprotected peptides, potentially bringing peptide stapling to a larger community than hitherto have had access and extends the applications of dibromomaleimide and related reagents. Future efforts will apply this approach to a wider range of helix mediated PPIs with the aim of establishing the generality of the method. The approach may also be useful to identify transient binding pockets proximal to a native ligand binding site using the template directed approach introduced by Sharpless.^70^ Similarly, the reversibility of the constraint, may permit more facile delivery of a peptide-based reagent to the cell, where it can become unconstrained and less easily transported back out of the cell. Together with the additional functional handle introduced on the maleimide constraint (which is orthogonal in terms of its reactivity), this could facilitate detailed cellular and biochemical analyses *e.g.* identification of additional targets of the constrained peptide by pull-down. These objectives will represent the focus of our future studies.
