# Supplementary material for: Double quick, double click reversible peptide “stapling”
Source: Chem Sci. 2017 May 31;8(7):5166–71. doi: 10.1039/c7sc01342f (PMC5618791; doi:10.1039/c7sc01342f)
Supplement: Supplementary file 1 [file SC-008-C7SC01342F-s001.pdf]

Electronic Supplementary Material (ESI) for Chemical Science.
This journal is © The Royal Society of Chemistry 2017

Double Quick, Double Click Reversible Peptide "Stapling"

Supporting Information

Claire M. Grison,[a],[b] George M. Burslem,[a],[b] Jennifer A. Miles,[a],[b] Ludwig K. A. Pilsl,[a],[b] David J. Yeo,[a],[b] Zeynab Imani,[a],[b] Stuart L. Warriner,[a],[b] Michael E. Webb[a],[b] and Andrew J. Wilson,*[a],[b]

[a] School of Chemistry, University of Leeds, Woodhouse Lane, Leeds LS2 9JT, UK.
[b] Astbury Centre For Structural Molecular Biology, University of Leeds, Woodhouse Lane, Leeds LS2 9JT, UK
E-mail: A.J.Wilson@leeds.ac.uk

General Comments **3**

Synthesis of S peptides **4**
- **WT-S peptide – 8** **5**
- **OX-S-Cys peptide – 2c** **6**
- **OX-S-hCys peptide – 2d** **7**

General procedure for the synthesis of reversible constrained peptides **8**
- **Synthesis of RED-peptides from OX-peptides - STEP 1** **8**
- **Synthesis of STA-peptides - STEP 2** **8**
- **Synthesis of OX-peptides from STA-peptides - STEP 3** **8**
- **HRMS spectra** **9**
  - BID-Cys - a 9
  - BID-hCys - b 10
  - S-Cys - c 11
  - S-hCys - d 12

Modulation of the constraint **13**
- **Synthesis of OX-BID-hCys 2b from STA-BID-hCys – 3b** **13**
- **Synthesis of the non-reversible constrained peptide - Suc-STA-S-hCys – 9** **14**

Synthesis of clicked constrained peptides **16**
- **Synthesis of *N*-propargyl-2,3-dibromomaleimide – 5** **16**
- **Synthesis of azide clickable intermediates I - VI** **16**
  - tert-butyl-(3-bromopropyl)carbamate – I 16
  - tert-butyl-(3-azidopropyl)carbamate – II 17
  - N-(3-azidopropyl)-2,2,2-trifluoroacetamide – III 17
  - 5-(3-(3-azidopropyl)thioureido)-2-(6-hydrodroxy-3-oxo-3H-xanthen-9-yl)benzoic acid – IV 17
  - N-(3-azidopropyl)-5-((3aR,4R,6aS)-2-oxohexahydro-1H-thieno[3,4-d]imidazol-4-yl)pentanamide – V 19

Click chemistry on stapled peptides **20**
  - Synthesis of yne-STA-BID-Cys – 6 20
  - Synthesis of biotinylated STA-BID peptide by click chemistry – 7a 21

Synthesis of fluorescein conjugated STA-BID by click chemistry – 7b 22
Synthesis of pegylated STA-BID peptide by click chemistry – 7c 23

Determination of the concentrations of purified peptides 25
Synthesis of maleimide(glutathione)$_2$ – VII 25
Molar extinction coefficient of maleimide crosslink using VII 27

Proteolysis studies on BID peptides 28
LCMS traces of RED-BID-Cys 1a in presence of Trypsin 29
LCMS traces of STA-BID-Cys 3a in presence of Trypsin 30
LCMS traces of RED-BID-hCys 1b in presence of Trypsin 31
LCMS traces of STA-BID-hCys 3b in presence of Trypsin 32

Circular Dichroism 33

Molecular Modelling 35
Molecular Mechanics calculation 35
Molecular Dynamics simulation 36
Comparison between RED-BID-hCys 1b and STA-BID-Cys 3a 36
Comparison between WT-S 8, STA-S-Cys 3c and STA-S-hCys 3d 38

Fluorescence Anisotropy 41
Competitive assay 43
Direct binding assay 44

RNA degradation assay 45
Preparation of S protein 45
Conditions of the assay 45

Bibliography 46

General Comments

All amino acids and resins were purchased from either Novabiochem (Merck) or Sigma-Aldrich. All amino acids were *N*-Fmoc protected and side chains were protected with Boc (Lys, Trp); O*t*Bu (Asp, Glu, Ser, Thr); Trt (Cys, Asn, Gln); Pbf (Arg). BID-Cys and BID-hCys peptides were purchased from Severn Biotech Ltd. Syntheses of peptides were carried out in the Liberty CEM Peptide Synthesiser with or without microwave assistance. All solvents used were of ACS grade from Sigma-Aldrich.

UV absorbance analyses were recorded in a 1 mm path-length quartz cuvette on a Perkin Elmer Lambda 900 UV/VIS/NIR spectrometer.

High-resolution mass spectrometry (HRMS) data was recorded using electrospray ionization in positive mode (ESI^+) with a Bruker MaXis Impact spectrometer.

LC-MS analyses were conducted on a Thermo Scientific Ultimate 3000 using $UHPLC^+$ technology. All experiments were run through a Kinetex C-18 50x2.1 mm LC-Column, 2.6 μm particle size, for small molecules using a 5-95% gradient (acetonitrile:water, 0.1% formic acid) over 1.7 min and an Aeris Peptide XB-C18 100x2.1 mm LC-Column, 2.6 μm particle size, for peptides using a 5-95% gradient (acetonitrile:water, 0.1% formic acid) over 3.6 min for positive ion spectra. Samples were ionized by electrospray.

Analytical HPLC experiments were performed using an Agilent 1290 Infinity LC series system equipped with an Ascentis Express Peptide ES-C18 100x2.1 mm column, 2.7 μm particle size on a 5-95% gradient (acetonitrile:water, 0.1% formic acid) over 5 min.

CD data were recorded using an Applied Photophysics Chirascan Instrument and Software in a 1 mm path-length quartz cuvette. For each scan, the following parameters were used: 180-260 nm range; point time 1 s; 1 nm per point; step = 1; bandwidth 5 nm; temperature 20 °C. Scans were performed in triplicate.

Fourier-transform infrared absorption spectroscopy (IR) was performed on Bruker Platinum-ATR system equipped with an Alpha FT-IR spectrometer. Maximum absorbances are reported for significant bands in cm^{-1}.

Synthesis of S peptides

The series of S peptides (WT-S **8**, OX-S-Cys **2c** and OX-S-hCys **2d**) (1 eq., 0.1 mmol) were synthesized on Rink Amide MBHA resin (Loading Capacity 0.36 mmol g^{-1}) using HCTU and DIPEA in DMF for amide coupling, and 20% piperidine in DMF for the deprotection using a Liberty CEM Peptide Synthesiser without microwave assistance. Each amino acid was coupled twice for 30 min at room temperature.

After the final amino acid coupling and deprotection, a solution of acetic anhydride (10 equiv.) and DIPEA (10 equiv.) in DMF (2 mL) was manually transferred to the resin. After 2 h, the resin was drained, washed with DMF (5 × 2 mL × 2 min), CH_2Cl_2 (5 × 2 mL × 2 min) and then Et_2O (3 × 2 mL × 2 min).

Peptides were simultaneously cleaved from the resin and side-chain deprotected with cleavage 'Reagent K' TFA:EDT:Thioanisole:Phenol:H_2O, 82:3:5:5:5 (3 × 2 mL × 1 h). The resin was washed with fresh TFA (2 mL × 2 min) and the solution concentrated *in vacuo*. The resulting oils were precipitated with ice-cold ether (5 mL) and placed in a centrifuge (3000 rpm × 3 min). The supernatants were removed, the precipitate rinsed with ice-cold ether (3 × 10 mL) and dried in vacuo.

Crude peptides were suspended in dimethylsulfoxide at an approximate concentration of 10 mg mL^{-1}. Purification was performed using a mass-directed HPLC with a Jupiter Proteo preparative column on a gradient of 20-45% acetonitrile/water (with 0.1% formic acid) for 35 min at a flow rate of 10 mL min^{-1} with S peptides eluting at 16-18 minutes. Fractions were checked by LCMS, concentrated *in vacuo* and lyophilised.

WT-S peptide – 8

The compound **8** was obtained using mass-directed preparative HPLC with purity higher than 95% (5.2 mg, 38%).

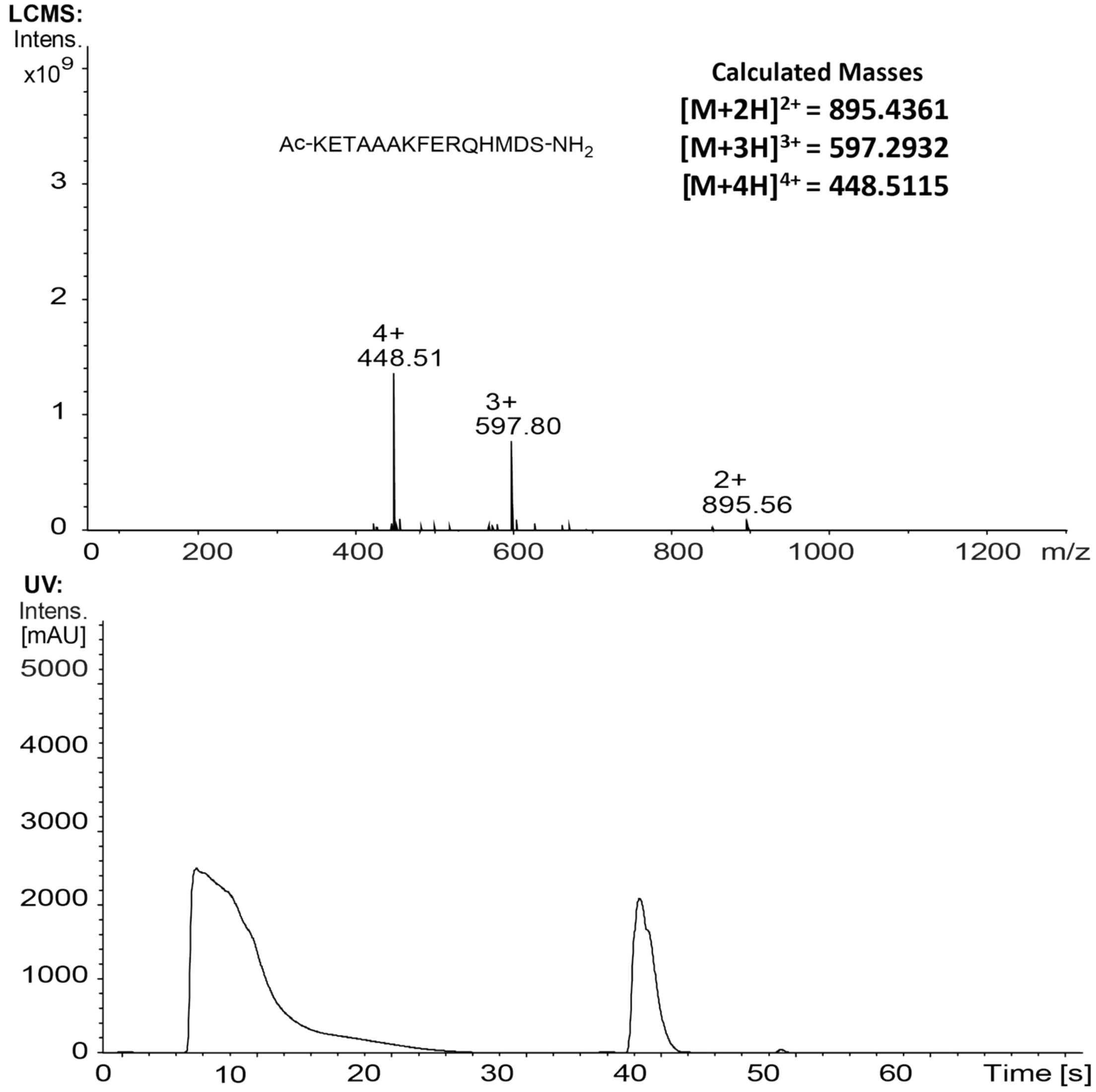

Figure S 1: Characterisation of WT-S peptide. A: MS traces; B: UV-LCMS trace (200-300 nm).

OX-S-Cys peptide – 2c

The compound **2c** was obtained using mass-directed preparative HPLC with purity higher than 95% (6.3 mg, 27.1%).

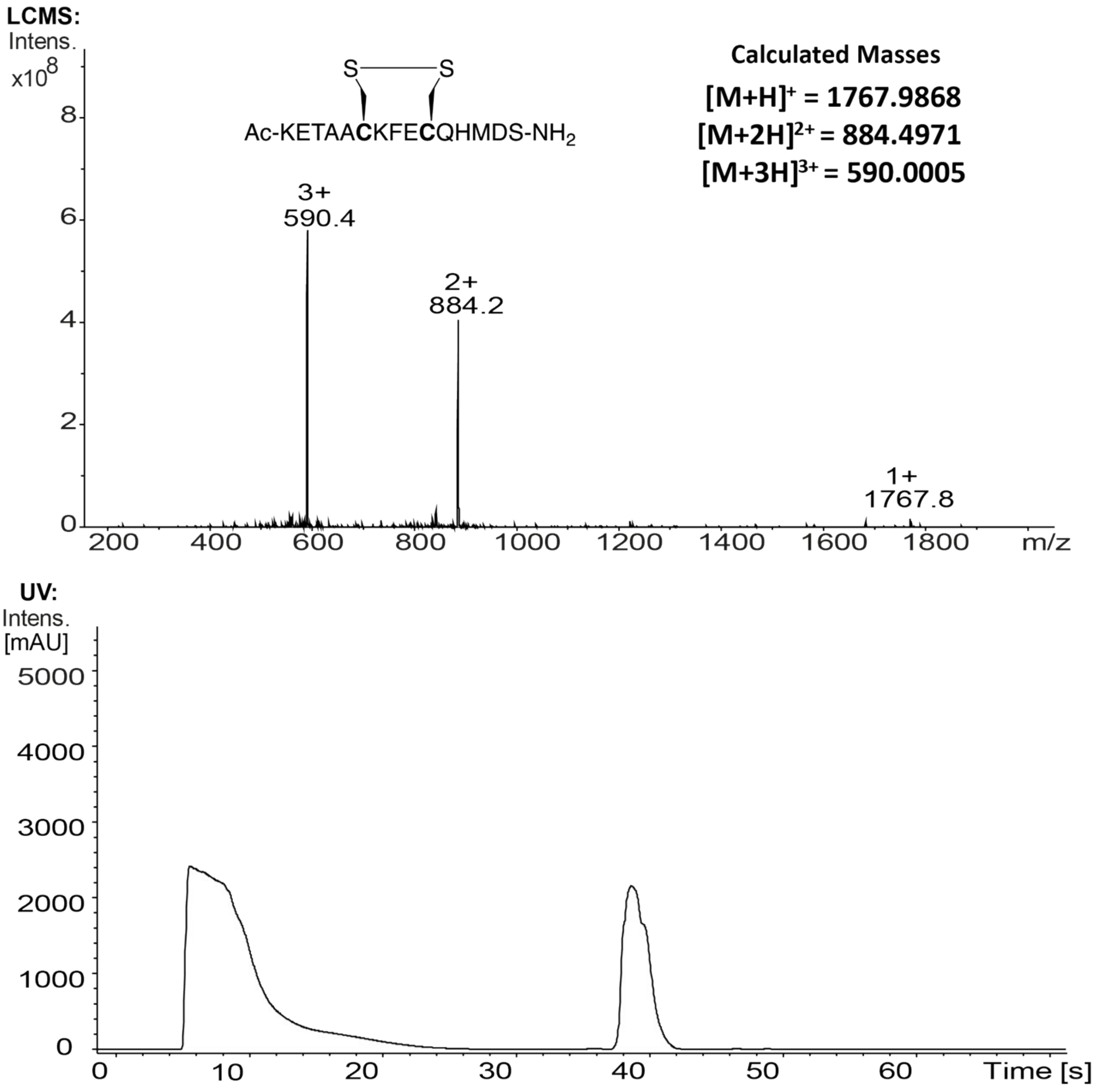

Figure S 2: Characterisation of OX-S-Cys peptide 2c. A: MS traces; B: UV-LCMS traces (200-300 nm).

OX-S-hCys peptide – 2d

The compound **2d** was obtained using mass-directed preparative HPLC with purity higher than 95% (8.1 mg, 22.2%).

LCMS:

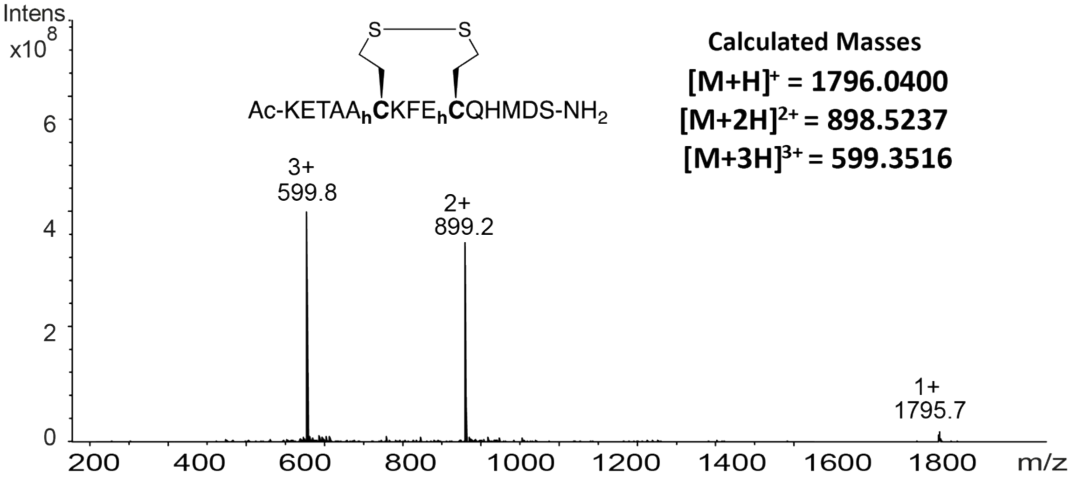

UV:

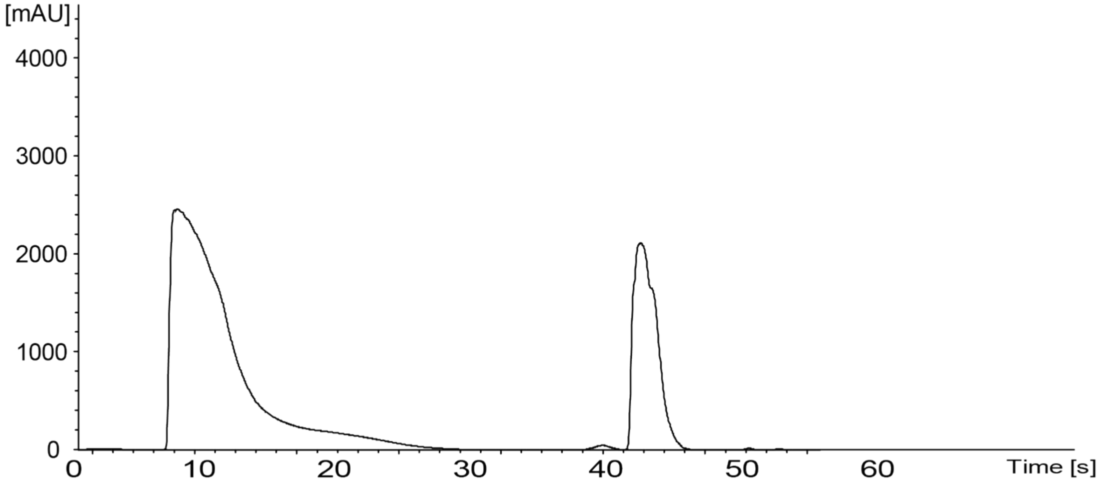

Figure S 3: Characterisation of OX-S-hCys peptide 2d. A: MS traces; B: UV-LCMS traces (200-300 nm).

General procedure for the synthesis of reversible constrained peptides

Synthesis of RED-peptides from OX-peptides - STEP 1

A solution of OX-peptide (1 eq.) in a mixture of water/acetonitrile (1 mL/0.1 mL per 0.8 μmol) was prepared in a 5 mL vial, to which was added a 10 mM solution of TCEP·HCl (1 eq.) in acetonitrile. The resulting mixture was stirred for 30 minutes at room temperature until completion; the reaction was monitored by HRMS.

Synthesis of STA-peptides - STEP 2

From the crude solution, or a fresh solution of RED-peptide (1 eq.) in a mixture of water/acetonitrile (1 mL/0.1 mL per 0.4 μmol) in a 5 mL vial, was added a 10 mM solution of dibromomaleimide (1 eq.) in acetonitrile. The resulting mixture was stirred for a few hours at room temperature until completion; the reaction was monitored by HRMS. The crude solution was directly injected onto the mass directed HPLC for purification. The desired peptide was collected by mass using a Jupiter Proteo preparative column (reversed phase) on an increasing gradient of acetonitrile to water (plus 0.1% formic acid v/v in both solvents) at a flow rate of 10 $mL.min^{-1}$.

Synthesis of OX-peptides from STA-peptides - STEP 3

From the crude solution of STA-peptide (1 eq.) in a mixture of water/acetonitrile (1 mL/0.1 mL per 0.4 μmol) in a 5 mL vial was added an excess of thiol* (26 eq.). The resulting mixture was stirred overnight and the reaction was monitored by HRMS.

*thiol: β-mercaptoethanol, glutathione.

HRMS spectra

BID-Cys – a

Each step of this series of reactions has a quantitative conversion. STA-BID-Cys **2a** was further purified by mass-directed HPLC for biophysical assay with purity higher than 95% (1.2 mg, 52%).

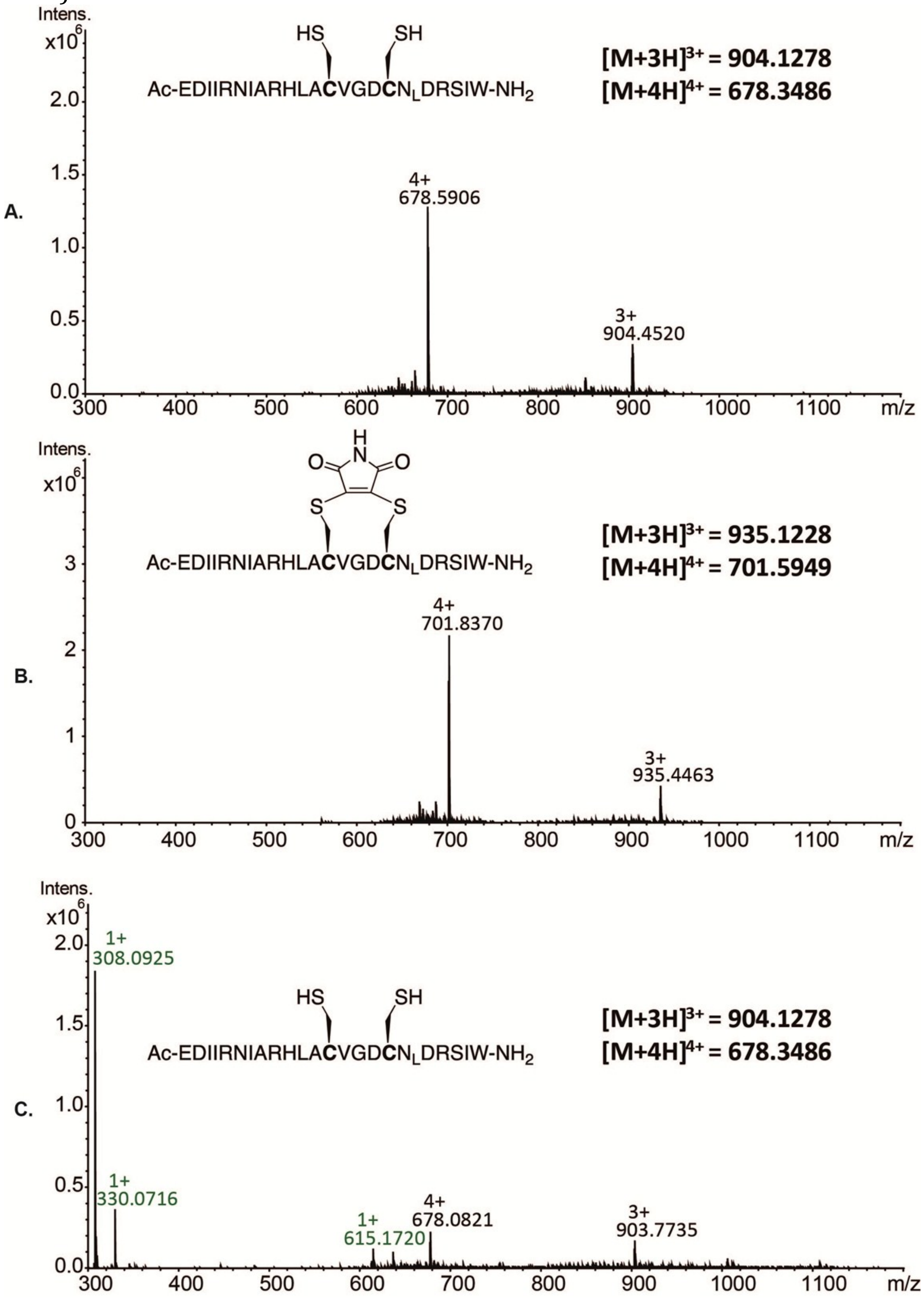

Figure S 4: HRMS spectra illustrating each step of the reversible constraint synthesis of BID-Cys. A to B: STEP 2; B to C: STEP 3. In graph C, the masses highlighted in green correspond to the excess of glutathione ($[M+H]^+$ = 308.0925; $[M+Na]^+$ = 330.0716; $[2M+H]^+$ = 615.1720).

BID-hCys – b

Each step of this series of reactions has a quantitative conversion. STA-BID-hCys **2b** was further purified by mass-directed HPLC for biophysical assay with purity higher than 95% (2.1 mg, 47%).

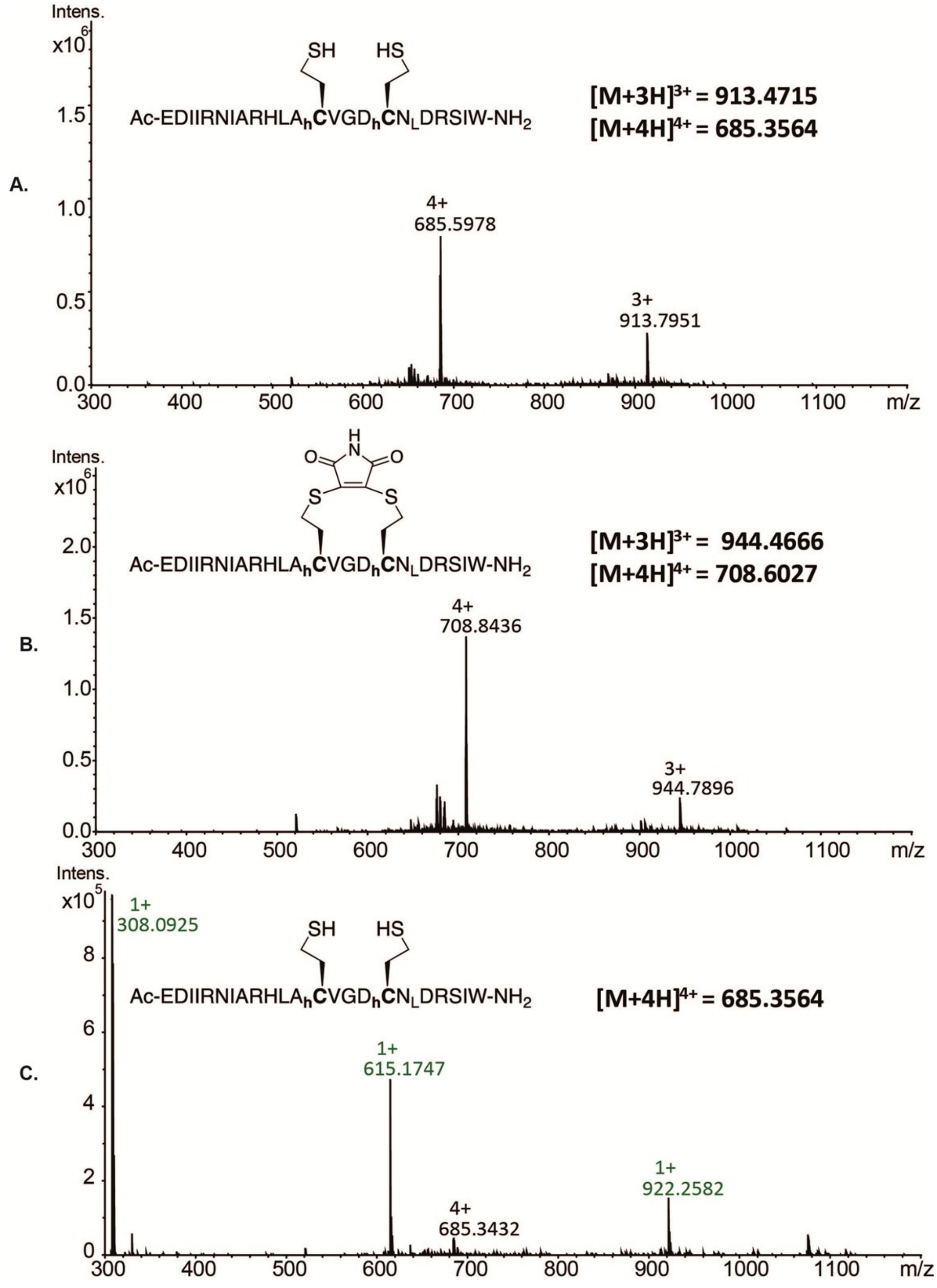

Figure S 5: HRMS spectra illustrating each step of the reversible constraint synthesis of BID-hCys. A to B: STEP 2; B to C: STEP 3. In graph C, the masses highlighted in green correspond to the excess of glutathione ($[M+H]^+$ = 308.0925; $[2M+H]^+$ = 615.1747; $[3M+H]^+$ = 922.2582).

S-Cys – c

Each step of this series of reactions has a quantitative conversion. STA-S-Cys **2c** was further purified by mass-directed HPLC for biophysical assay with purity higher than 95% (0.97 mg, 65%).

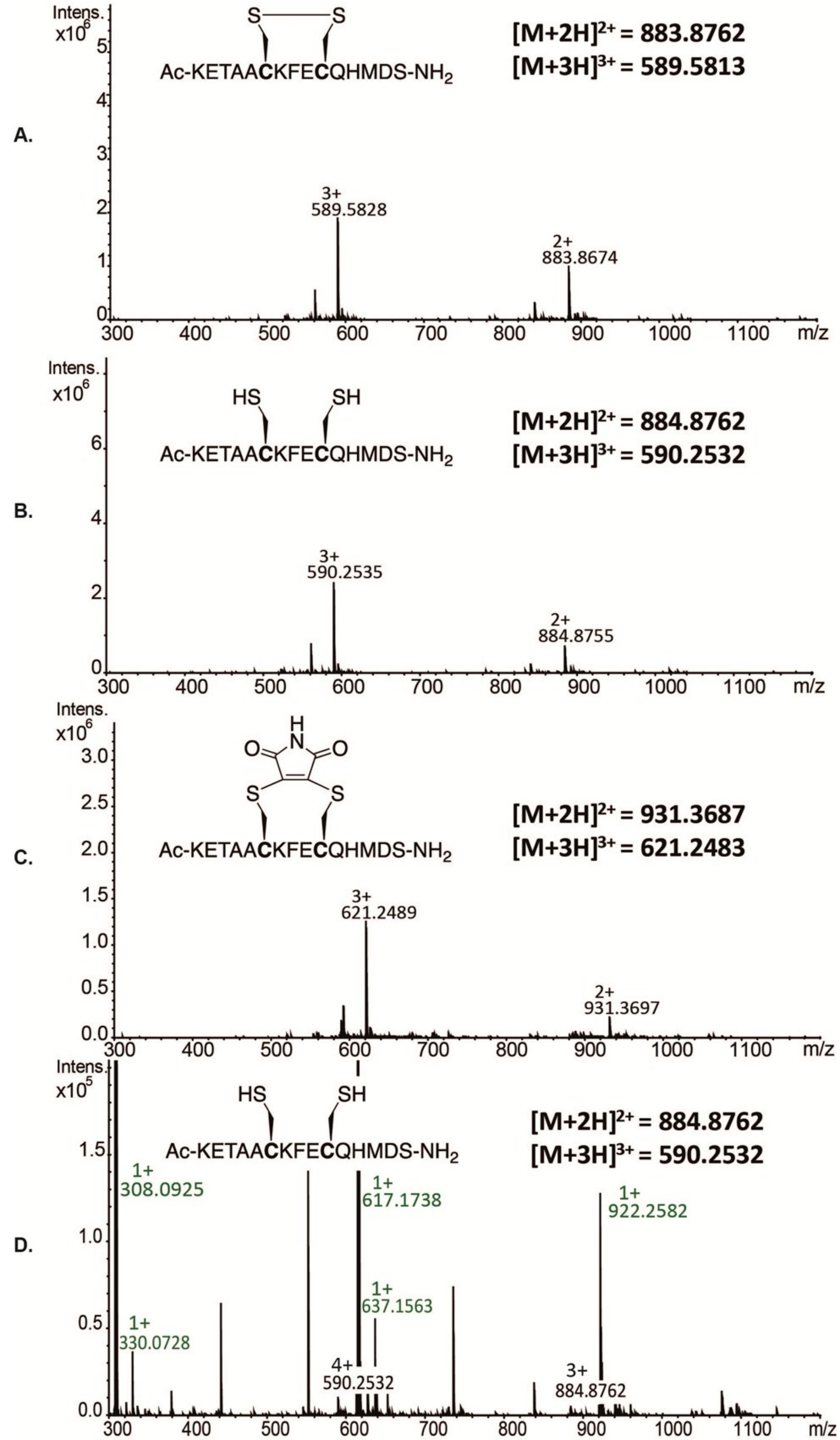

Figure S 6: HRMS spectra illustrating each step of the reversible constraint synthesis of S-Cys peptide. A to B: STEP 1; B to C: STEP 2; C to D: STEP 3. In graph D, the masses highlighted in green correspond to the excess of glutathione ($[M+H]^+$ = 308.0925; $[M+Na]^+$ = 330.0728; $[2M+H]^+$ = 615.1738, [2M+Na]+ = 637.1563; $[3M+H]^+$ = 922.2582).

S-hCys – d

Each step of this series of reactions has a quantitative conversion. STA-S-hCys **2d** was further purified by mass-directed HPLC for biophysical assay with purity higher than 95% (1.1 mg, 56%).

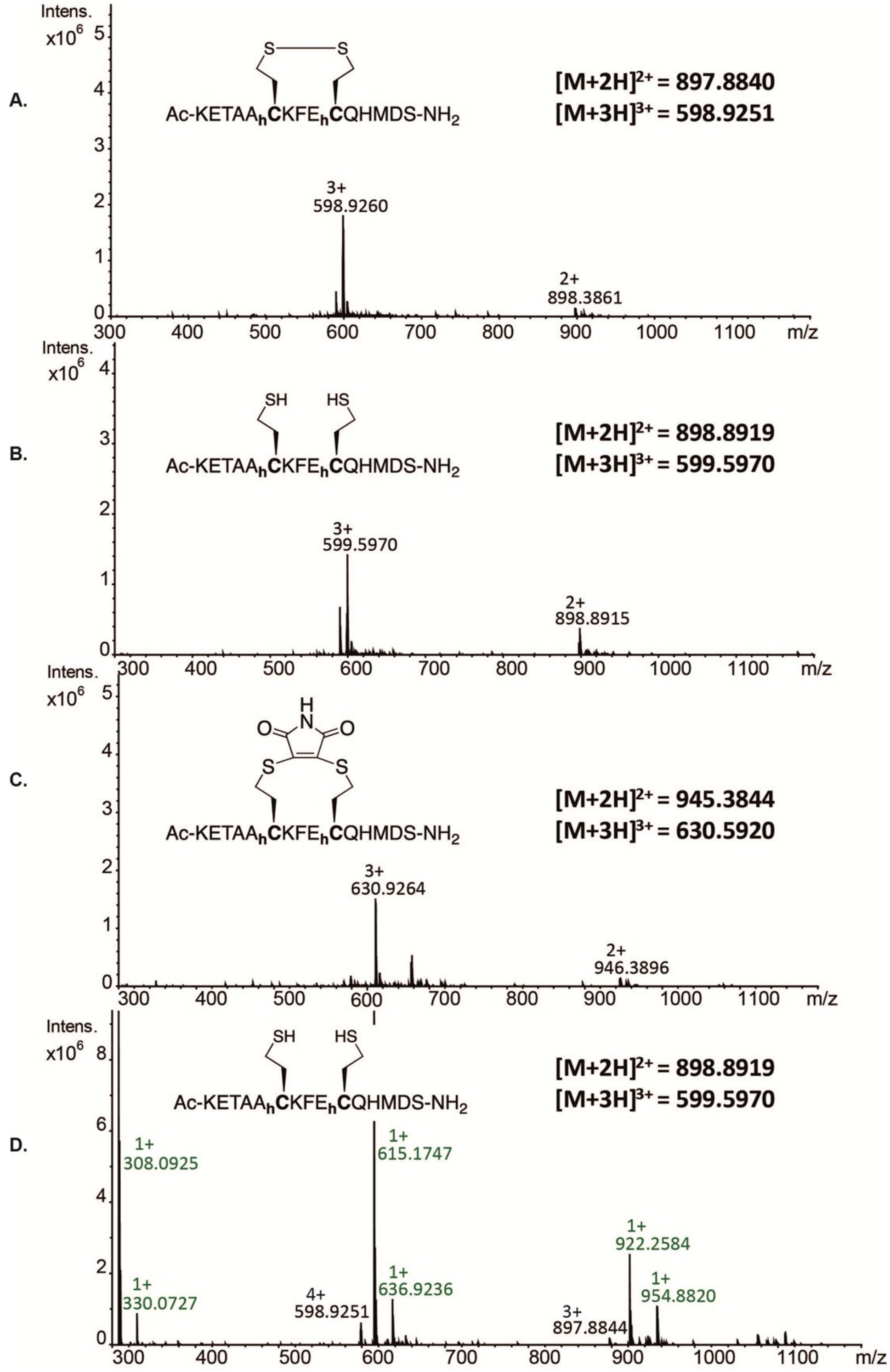

Figure S 7: HRMS spectra illustrating each step of the reversible constraint synthesis of S-hCys peptide. A to B: STEP 1; B to C: STEP 2; C to D: STEP 3. In graph D, the masses highlighted in green correspond to the excess of glutathione ([M+H]$^+$ = 308.0925; [M+Na]$^+$ = 330.0727; [2M+H]$^+$ = 615.1747, [2M+Na]+ = 636.9236; [3M+H]$^+$ = 922.2584, [3M+Na]$^+$ = 954.8820).

Modulation of the constraint

Synthesis of OX-BID-hCys 2b from STA-BID-hCys – 3b

The STA-peptides can be unconstrained to give the RED-peptides and constrained again through a disulphide bond giving the OX-peptides.

Oxygen was directly bubbled into the crude solution of RED-BID-hCys **1b** (1 eq., 1.1 mg, 0.4 μmol) in a mixture of water/acetonitrile (1 mL/0.1 mL) in a 5 mL vial for 5 min to give OX-BID-hCys **2b**. The reaction was monitored by LCMS (Figure S 8).
Each step of this series of reactions has a quantitative conversion.

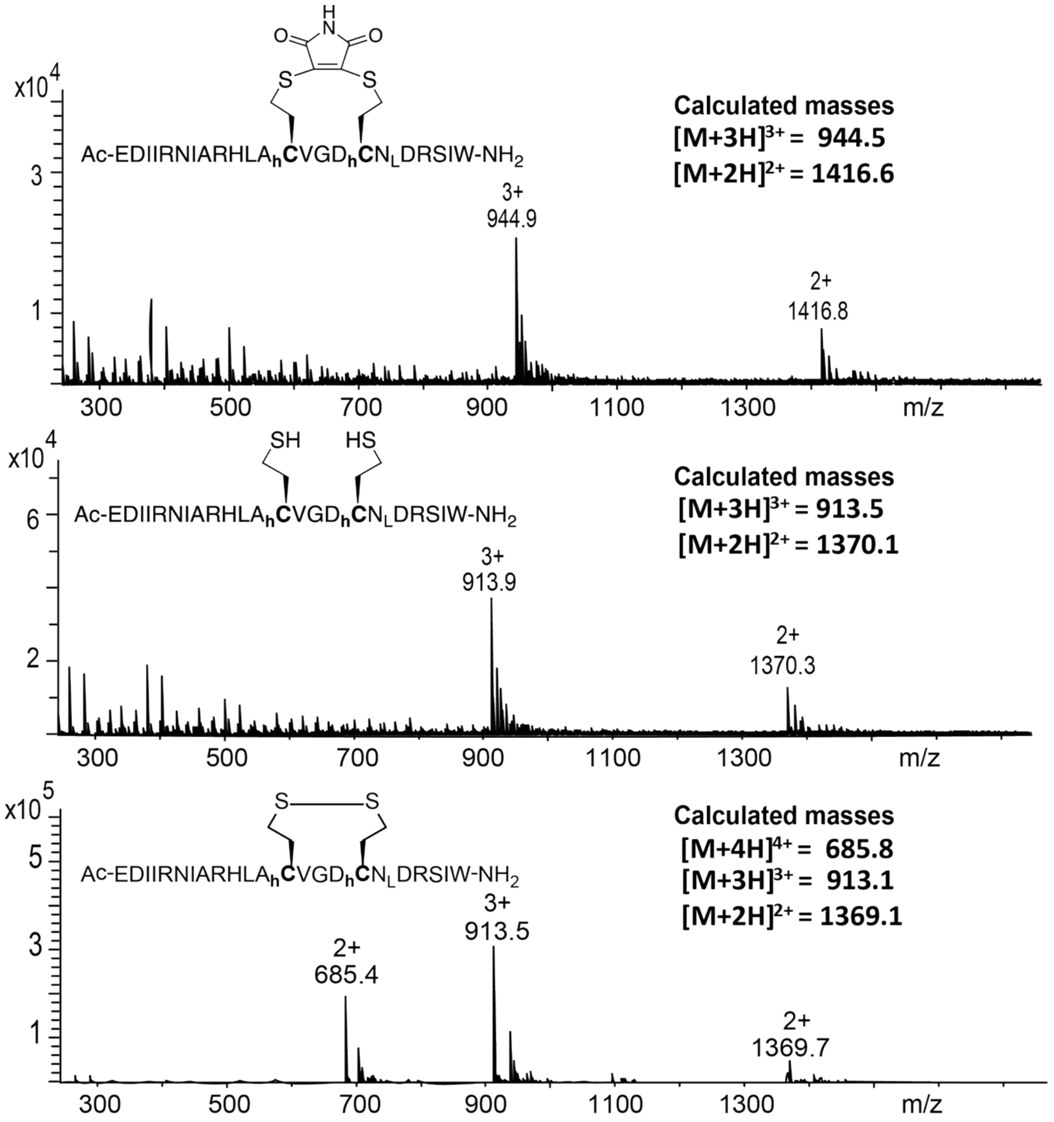

Figure S 8: LCMS spectra illustrating each step of the reversible constraint synthesis of BID-hCys peptide. A to B: STEP2; B to C: STEP 4.

Synthesis of the non-reversible constrained peptide - Suc-STA-S-hCys – 9

The RED-peptides can be irreversibly constrained by adding using dibromomaleimide with an excess of reducing agent, leading to a succinimide cross-link.

From the crude solution solution of RED-S-hCys **1d** (1 eq., 2 mg, 1.13 μmol) in a mixture of water/acetonitrile (1.6 mL/ 0.4 mL) in a 5 mL vial, was added a 10 mM solution of dibromomaleimide (1 eq., 170 μL, 1.13 μmol) in acetonitrile. The resulting mixture was stirred for 2 hrs at room temperature. TCEP·HCl (5 eq., 1.6 mg, 5.65 μmol) was then added to give in 8 hrs Suc-STA-S-hCys **9**. An excess of thiol* (26 eq.) was added to the reaction mixture and Suc-STA-S-hCys **9** remained intact; the reaction was monitored by HRMS (Figure S 9).

*: β-mercaptoethanol (1.9 μL, 29.4 μmol), or glutathione (9.0 mg, 29.4 μmol)

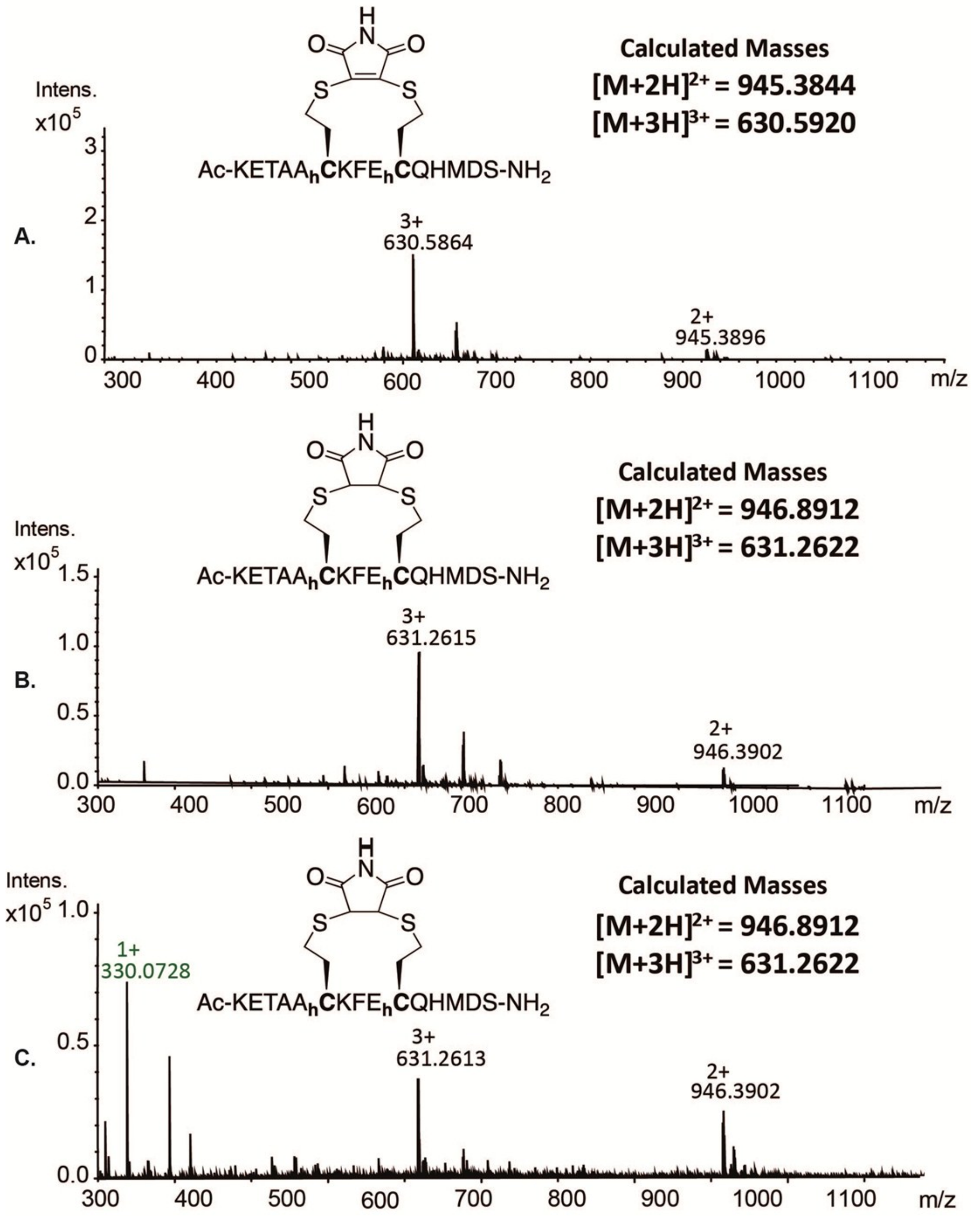

Figure S 9: HRMS spectra illustrating each step of the synthesis of non-reversible constrained synthesis of Suc-STA-S-hCys 9; the mass highlighted in green corresponds to an excess of glutathione: ($[M+Na]^+$ = 330.0727; $[2M+H]^+$)

Synthesis of clicked constrained peptides

Synthesis of *N*-propargyl-2,3-dibromomaleimide – 5

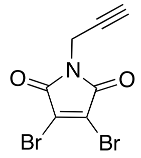

PPh_3 (1.23 g, 4.72 mmol, 1.2 eq.) was dissolved in anhydrous and degassed THF (50 mL) and cooled to 0 °C. DEAD (743 µL, 4.72 mmol, 1.2 eq.) was added to the mixture and was stirred at 0 °C for 10 minutes. Propargyl alcohol (350 µL, 6.01 mmol, 1.5 eq.) and dibromomaleimide (1 g, 3.94 mmol, 1 eq.) were sequentially added and the reaction was allowed to warm to room temperature with stirring for 20 hrs. The reaction mixture was then concentrated under reduced pressure and the residue purified by flash chromatography (Et_2O/hexane : 10/90) to give **5** as a colourless solid (674 mg, 2.3 mmol, 58%). R_f 0.70 (9/1 : PE/Et_2O); ^1H NMR (500 MHz, $CDCl_3$) δ 2.24 (t, C*H*≡C, J = 2.5 Hz, 1H), 4.33 (d, CH_2, J = 3 Hz, 2H); ^{13}C NMR (125 MHz, $CDCl_3$) δ 28.6 (CH_2), 72.5 (H*C*≡C-C), 76.1 (*C*≡*C*-C), 129.8 (*C*=C), 162.6 (*C*=O); IR (DMSO): ν_{max} 2130 (*C*≡C).

The spectral data were identical to those reported previously.[1]

Synthesis of azide clickable intermediates I - VI

***tert-butyl-(3-bromopropyl)carbamate* – I**

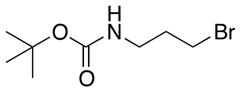

To a cooled suspension of 3-bromopropylamine hydrobromide (5 g, 22.8 mmol, 1 eq.) and di-*tert*-butyl dicarbonate (5 g, 22.8 mmol, 1 eq.) in CH_2Cl_2 (11 mL), triethylamine (6.4 mL, 45.6 mmol, 2 eq.) was added dropwise. The reaction mixture was stirred overnight at room temperature. After wich the reaction mixture was washed with a 1 M solution of HCl (2 x 10 mL) and H_2O (2 x 10 mL). The organic layer was dried over $MgSO_4$, filtrated and evaporated under reduced pressure affording the product as a yellow oil (3 g, 13.3 mmol, 58%). R_f 0.75 (8/2 : PE/EtOAc);^1H NMR (500 MHz, $CDCl_3$) δ 4.88 (bs, NH, 1H), 3.40 (t, CH_2Br, J = 6.4 Hz, 2H), 3.21 (bs, CH_2NH, 2H), 1.98-2.02 (m, CH_2, 2H), 1.39 (s, $C(CH_3)_3$, 9H).

The spectral data were identical to those reported previously.[2,3]

***tert-butyl-(3-azidopropyl)carbamate* – II**

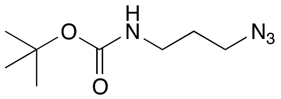

tert-Butyl (3-bromopropyl)carbamate (3.2 g, 13.3 mmol, 1 eq.) and sodium azide (1.7 g, 26.6 mmol, 2 eq.) were dissolved in a mixture of H_2O/dioxane (1/1, 10 mL). The reaction was heated to reflux at 75 °C for 2 hrs and stirred overnight at room temperature. The reaction was diluted with CH_2Cl_2 (15 mL) and washed with H_2O (3 x 10 mL). The organic layer was dried over $MgSO_4$, filtrated and evaporated under reduced pressure affording **II** as a pale yellow oil (1.5 g, 7.6 mmol, 57%). R_f 0.77 (8/2 : PE/EtOAc); ^{1}H NMR (500 MHz, $CDCl_3$) δ 4.80 (bs, *NH*, 1H), 3.28 (t, CH_2N_3, J = 7 Hz, 2H), 3.21 (t, CH_2NH, J = 5.5 Hz, 2H), 1.65-1.71 (m, CH_2, 2H), 1.36 (s, $C(CH_3)_3$, 9H).

The spectral data were identical to those reported previously.[2,3]

***N-(3-azidopropyl)-2,2,2-trifluoroacetamide* – III**

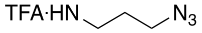

tert-Butyl-(3-azidopropyl)carbamate (3 g, 15 mmol, 1 eq.) was dissolved in anhydrous CH_2Cl_2 (23 mL). TFA (23 mL, 300 mmol, 20 eq.) was added and stirred for 4 hrs at room temperature. Then the excess TFA and CH_2Cl_2 were removed under reduced pressure affording the TFA salt **III** as a blurry oil (3.2 g, 15 mmol, quant.). R_f 0.25 (9:1 CH_2Cl_2:MeOH); ^{1}H NMR (500 MHz, $CDCl_3$) δ 8.22 (bs, *NH*, 1H), 3.57 (bs, CH_2N_3, 2H), 3.17 (bs, CH_2NH, 2H), 2.10 (bs, CH_2, 2H).

Special care was taken considering this product could be potentially explosive. It was stored at 0 °C, never heated over 40 °C and any impact or grinding with metal was avoided.

The spectral data were identical to those reported previously.[2,3]

***5-(3-(3-azidopropyl)thioureido)-2-(6-hydrodroxy-3-oxo-3H-xanthen-9-yl)benzoic acid* – IV**

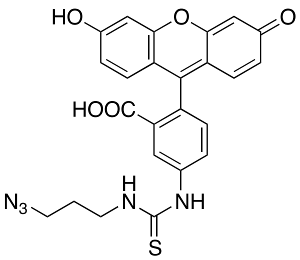

Fluorescein isothiocyanate isomer I (199 mg, 0.5 mmol, 1 eq.) was dissolved in anhydrous THF (20 mL) and triethylamine (171 µL, 1.2 mmol, 2.4 eq.) was added followed by the TFA salt **IV** (100 mg, 0.6 mmol, 1.2 eq.). The reaction mixture was stirred for 48 hrs at room temperature. THF was evaporated under redcued pressure and the crude residue was diluted in 50 mL of H_2O. The aqueous solution was slowly acidified with a concentrated solution of HCl at 0 °C and was extracted with EtOAc (5 x 60 mL). The organic layers were dried over $MgSO_4$, filtrated and evaporated affording the product as an orange solid (200 mg, 0.4 mmol, 82 %). The residue was further purified by HPLC using a gradient of CH_3CN/H_2O from 95/5 to 5/95 for 15 min. R_f 0.78 (9/1 : CH_2Cl_2/CH_3OH); ^1H NMR (500 MHz, CD_3OD) δ 8.07 (d, O=C-C*H*=C, J = 1.6 Hz, 1H), 7.71 (dd, O=C-C*H*=CH, J = 1.6 Hz, J = 8.2 Hz, 1H), 7.12 (d, O=C-CH=C*H*, J = 8.2 Hz, 1H), 6.65 (d, ArC*H*, J = 8.7 Hz, 2H), 6.64 (d, ArC*H* , J = 2.3 Hz, 2H), 6.51 (dd, ArC*H*, J = 2.3 Hz, J = 8.7 Hz, 2H), 3.67 (t, CH_2N_3, J = 5.5 Hz, 2H), 3.40 (t, CH_2NH , J = 6.7 Hz, 2H), 1.90 (quint, CH_2, J = 6.6 Hz, 2H); LC-MS : m/z = 490.2; IR (DMSO) ν_{max} 2096.52 (N_3); HRMS (ESI): 490.1198 [M+H]+ found, $C_{24}H_{19}N_5O_5S$ calcd 490.1180.

The spectral data were identical to those reported previously.[2]

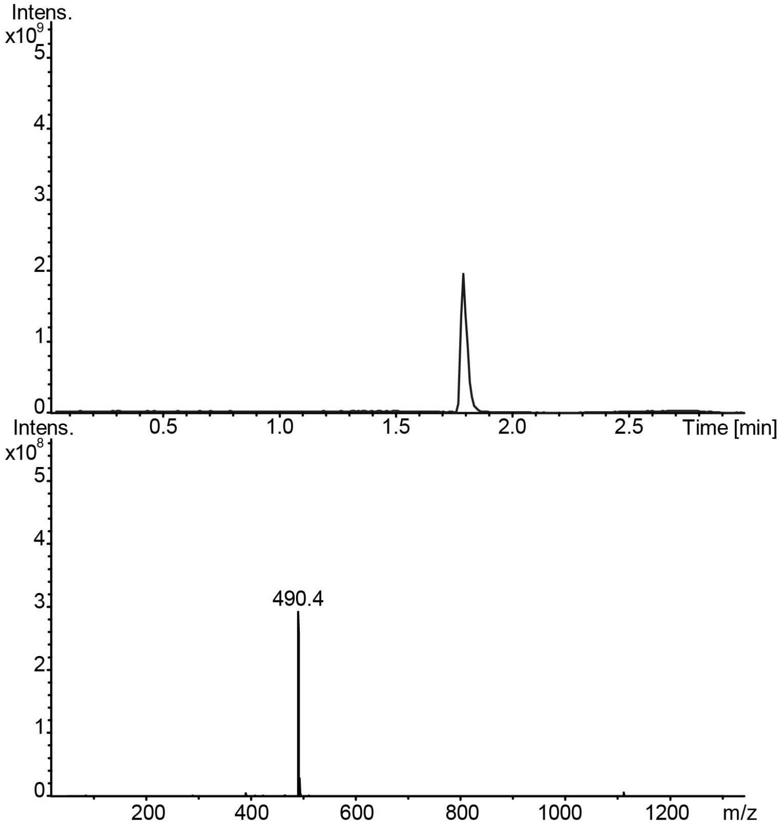

Figure S 10: Characterisation of IV. A: MS traces; B: UV-LCMS traces.

***N-(3-azidopropyl)-5-((3aR,4R,6aS)-2-oxohexahydro-1H-thieno[3,4-d]imidazol-4-yl)pentanamide* – V**

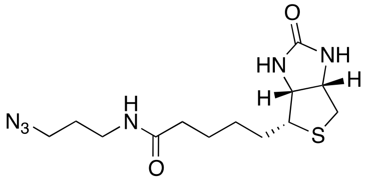

D-biotin (493 mg, 2.02 mmol, 1 eq.), HOBt (409 mg, 4.42 mmol, 2.2 eq.), EDCI (530 µL, 2.42 mmol, 1.2 eq.) and DIPEA (300 µL, 1.37 mmol, 0.7 eq.) were dissolved in anhydrous DMF (10 mL) and cooled to 0 °C. A solution of **III** (331 mg, 2.42 mmol, 1. 2 eq.) and DIPEA (400 µL, 1.83 mmol, 0.9 eq.) in anhydrous DMF (10 mL) was stirred for 5 min and was added to the activated D-biotin reaction mixture. The resulting mixture was stirred for 48 h at room temperature. DMF was evaporated under reduced pressure and in vacuo. The crude residue was purified by flash chromatography using a gradient of CH_2Cl_2/CH_3OH from 100/0 to 80/20 for 30 min. The residue was further purified by HPLC using a gradient of CH_3CN/H_2O from 95/5 to 5/95 for 20 min) to give **V** as a white solid (71 mg, 0.2 mmol, 11%). R_f 0.36 (9/1 : CH_2Cl_2/CH_3OH); ^1H NMR (500 MHz, CD_3OD) δ 3.67 (dd, C(3aS)*H*, J = 4.7 Hz, J = 7.7 Hz, 1H), 3.48 (dd, C(6aR)*H*, J = 4.5 Hz, J = 7.9 Hz, 1H), 2.53 (t, CH_2N_3, J = 6.9 Hz, 2H), 2.49 (dd, CH_2NH , J = 1.5 Hz, J = 5.0 Hz, 2H), 2.43 (t, CH_2C=O, J = 7.1 Hz, 2H), 2.38 (quad, C*H*-S, J = 4.5 Hz, 1H), 2.11 (dd, C*H*(Pro S)H-S, J = 6.0 Hz, J = 13.0 Hz, 1H), 1.89 (d, C*H*(Pro R)H-S, J = 12.6 Hz, 1H), 1.39 (dt, CH_2-CH_2-CH-S, J = 0.7 Hz, J = 7.5 Hz, 2H), 0.85 (m, CH_2CH_2 , J = 6.7 Hz, 2H), 0.62 (quint, CH_2, J = 7.6 Hz, 2H); LC-MS : m/z = 327.2; IR (DMSO) ν_{max} 2096.75 (N_3); HRMS (ESI): 327.1600 $[M+H]^+$, 653.3131 $[2M+H]^+$ found, $C_{13}H_{22}N_6O_2S$ calcd 327.1598.

Figure S 11: Characterisation of V. A: MS traces; B: UV-LCMS traces.

The spectral data were identical to those reported previously.[3]

Click chemistry on stapled peptides

***Synthesis of yne-STA-BID-Cys* – 6**

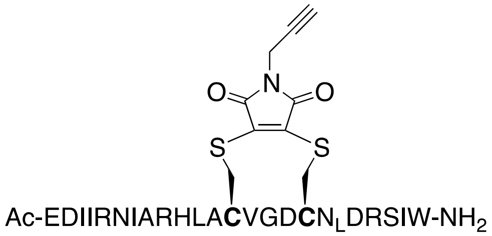

From the crude solution, or a fresh solution of OX-peptides (1 eq.) in a mixture of water/acetonitrile (1 mL/0.1 mL per 0.4 μmol) was prepared in a 5 mL vial, to which was added a 10 mM solution of *N*-propargyl-2,3-dibromomaleimide **5** (1 eq.) in acetonitrile. The resulting mixture was stirred for 20 minutes at room temperature until completion; the reaction was monitored by HRMS. The crude solution was evaporated under reduced pressure to afford the clickable peptide **6** (quantitative) as a yellow solid.

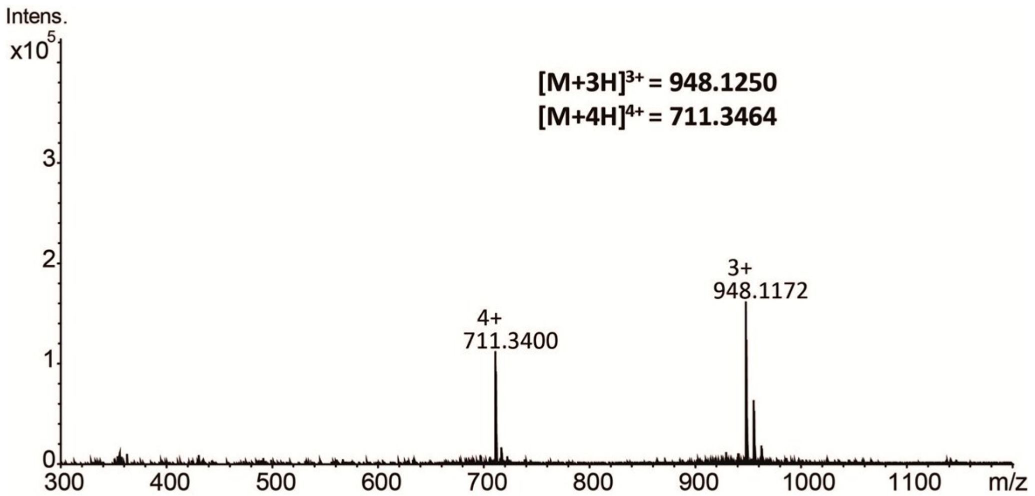

Figure S 12: HRMS trace of yne-STA-BID-Cys peptide 6.

***Synthesis of biotinylated STA-BID peptide by click chemistry* – 7a**

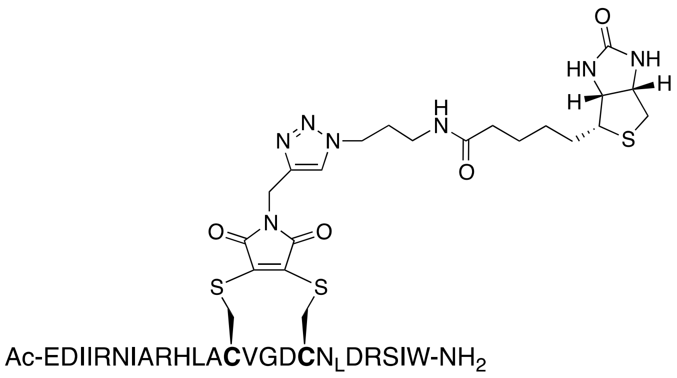

A solution of yne-STA-BID-Cys **6** (0.79 mg, 0.28 μmol, 1 eq.) was prepared in a 5 mM phosphate buffer (pH = 7.5) (78 μL) in a 1 mL vial, to which was added a 30 mM solution of biotin azide **V** (18 μL, 0.56 μmol, 2 eq.), a 50 mM solution of sodium ascorbate (3 μL, 0.14 μmol, 0.05 eq.) in water and a 50 mM solution of $CuSO_4{\cdot}5H_2O$ (1.1 μL, 0.05 μmol, 0.2 eq.) in water. The resulting mixture was heated to 50 °C for 1 min and stirred for 2 hrs at room temperature to afford **7a** (quantitative); the reaction was monitored by HRMS.

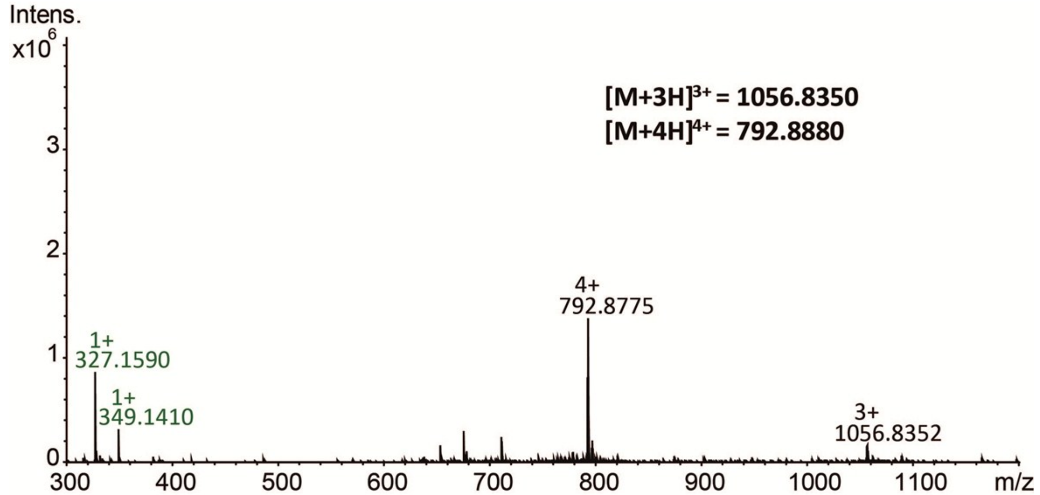

Figure S 13: HRMS trace of biotinylated STA-BID peptide 7a. The mass highlighted in green corresponds to the excess of biotine azide ([M+H]$^+$ = 327.1590, [M+Na]$^+$ = 349.1410).

***Synthesis of fluorescein conjugated STA-BID by click chemistry* – 7b**

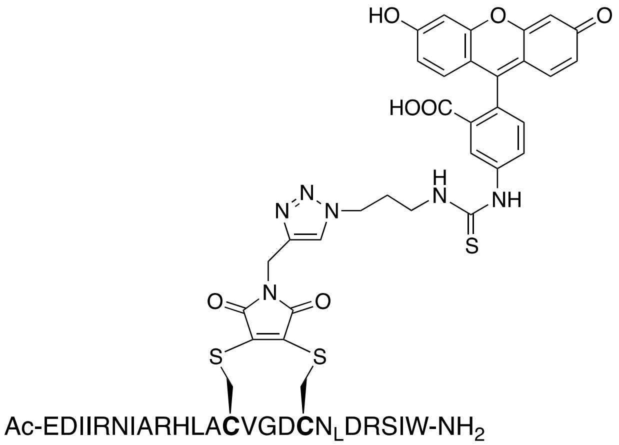

A solution of yne-STA-BID-Cys **6** (2.4 mg, 0.96 µmol, 1 eq.) was prepared in DMSO (100 µL) in a 1 mL vial, to which was added a 72 mM solution of FITC-Azide **IV** (51 µL, 3.84 µmol, 4 eq.) in DMSO, a 50 mM solution of CuI (20 µL, 0.96 µmol, 1 eq.) in CH_3CN, a 50 mM solution of sodium ascorbate (41 µL, 1.92 µmol, 2 eq.) in water and a 50 mM solution of DIPEA (20 µL, 0.96 µmol, 1 eq.) in water. The resulting mixture was stirred overnight at room temperature to afford **7b** (quantitative); the reaction was monitored by HRMS.

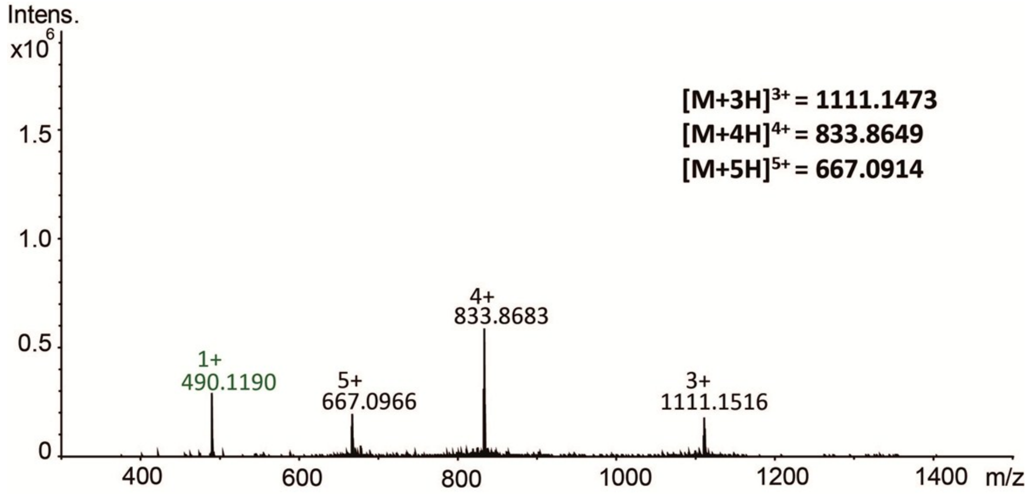

Figure S 14: HRMS trace of the fluorescein labelled STA-BID peptide 7b. The mass highlighted in green corresponds to the excess of FITC-Azide ([M+H]$^+$ = 490.1190).

***Synthesis of pegylated STA-BID peptide by click chemistry* – 7c**

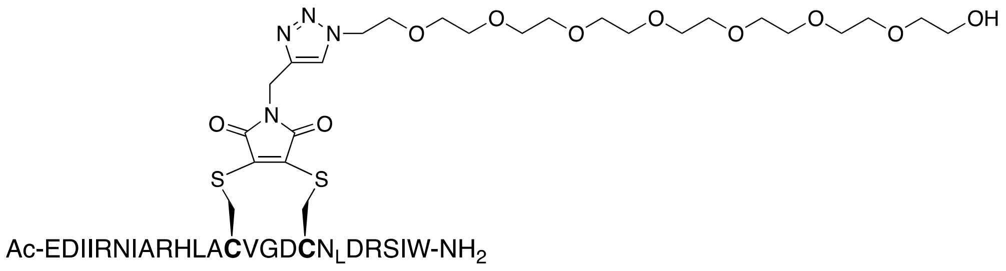

A solution of yne-STA-BID-Cys **6** (0.79 mg, 0.28 µmol, 1 eq.) was prepared in a 5 mM phosphate buffer (pH = 7.5) (78 µL) in a 1 mL vial, to which was added a 30.8 mM solution of *O*-(2-Azidoethyl)heptaethylene glycol (18 µL, 0.56 µmol, 2 eq.) in DMSO, a 50 mM solution of sodium ascorbate (3 µL, 0.14 µmol, 0.5 eq.) in water and a 50 mM solution of $CuSO_4 \cdot 5H_2O$ (1.1 µL, 0.05 µmol, 0.2 eq.) in water. The resulting mixture was heated to 50 °C for 1 min and stirred for 2 hrs at room temperature to afford **7c** (quantitative); the reaction was monitored by HRMS.

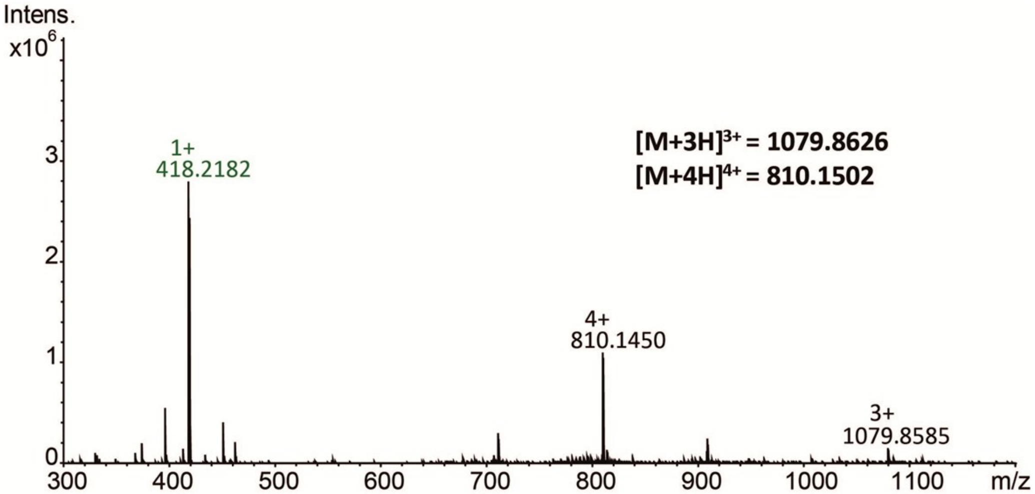

Figure S 15: HRMS trace of pegylated STA-BID peptide 7c. The mass highlighted in green corresponds to the excess of Azidoethyl)heptaethylene glycol ($[M+H]^+$ = 418.2182).

Determination of the concentrations of purified peptides

Peptides were lyophilised after purification by HPLC. The calculation of peptide concentration based on their weight was uncertain as lyophilised peptides might contain bound water. Peptide concentration can be accurately determined by UV absorbance based on the extinction coefficient of its residues.
A standard for the maleimide cross-link was needed to use in calculations of the constrained peptide extinction coefficient. The adduct between two molecules of glutathione and the maleimide cross-link was therefore synthesised on gram scale.

Synthesis of maleimide(glutathione)$_2$ – VII

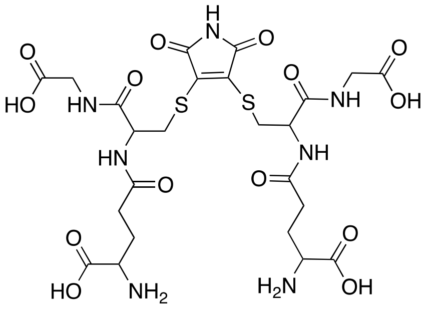

To a solution of glutathione (512 mg, 1.67 mmol, 2.5 eq.) in a mixture of water/acetonitrile (25 mL/ 25 mL) was added dibromomaleimide (169 mg, 0.67 mmol, 1 eq.). The resulting mixture was stirred for 4 h at room temperature; the reaction was monitored by HRMS. Solvents were evaporated under reduced pressure. The crude residue was dissolved in a minimum of water and was directly injected on the mass directed HPLC for purification. The desired maleimide adduct **VII** was collected by mass using a Jupiter Proteo preparative column (reversed phase) on a 5% to 95% gradient of acetonitrile to water (plus 0.1% formic acid v/v in both solvents) at a flow rate of 10 mL.min^{-1} (590 mg, 50 %). HRMS (ESI): 708.1602 $[M+H]^+$ found, $C_{24}H_{33}N_7O_{14}S_2$ calcd 708.1600.

LCMS:

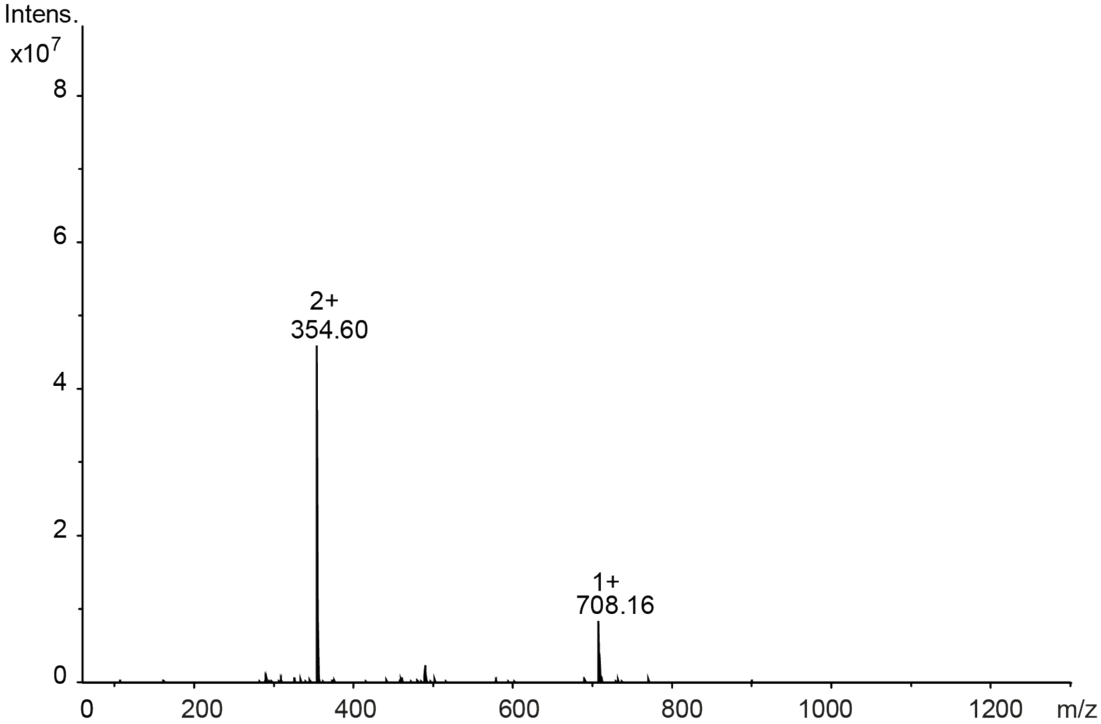

UV:

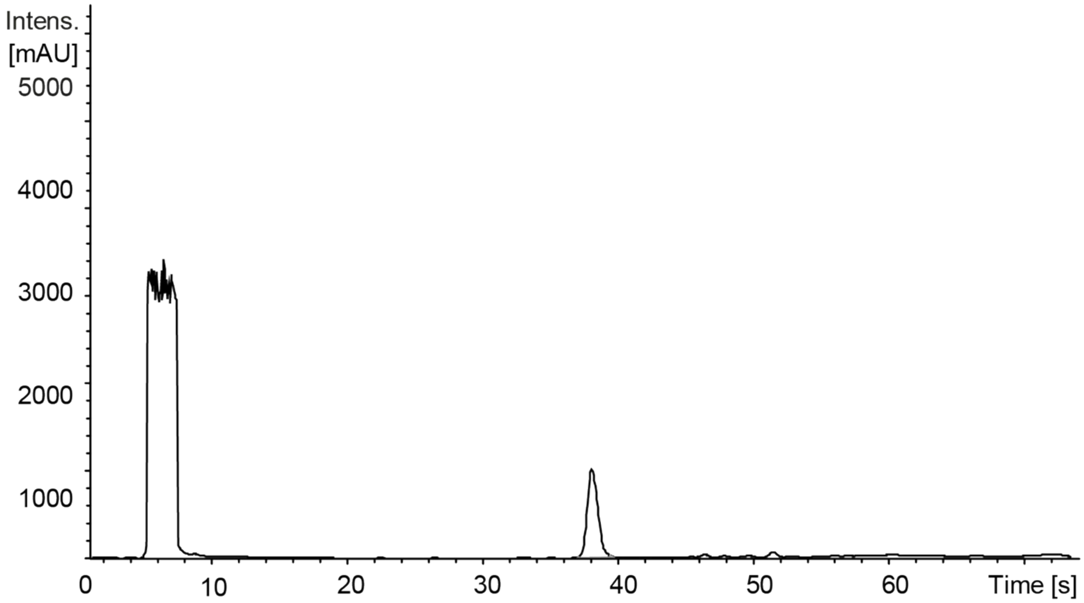

Figure S 16: Characterisation of maleimide(glutathione)$_2$ VII. A: MS traces; B: UV-LCMS traces.

Molar extinction coefficient of maleimide crosslink using VII

Solutions of **VII** were prepared at different concentrations: 500, 250, 125, 72.5, 36.25, 18 µM. The UV absorbance of the different solutions was recorded in 1 mm quartz cells from 500 nm to 250 nm.

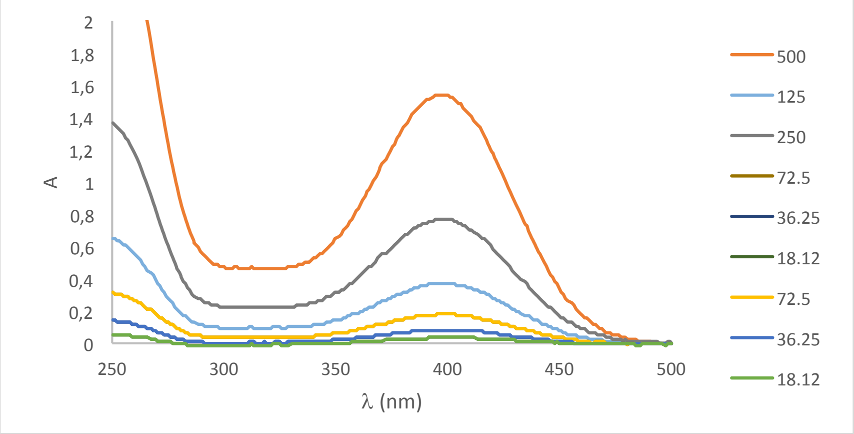

Figure S 17: UV absorbance of maleimide crosslink

The UV absorbance at 280 and 400 nm versus the concentration of maleimide adduct were plotted (Figure S 18). Following the Beer–Lambert law, a linear regression was applied and the molar extinction coefficient (λ) of maleimide cross-link was deduced.

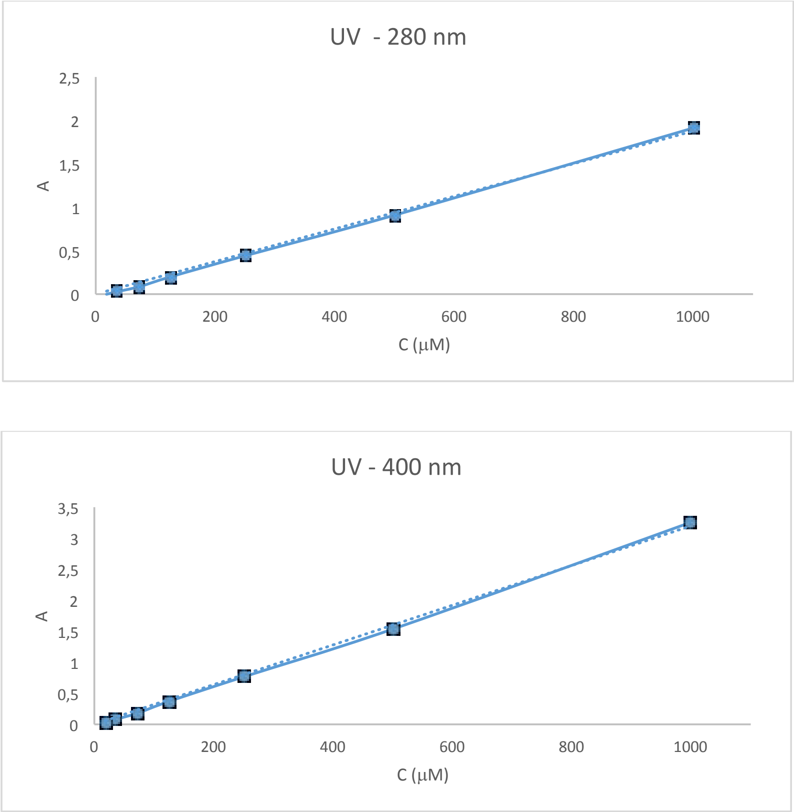

A.

$A = 0.0019*[C]$
$r^2 = 0.997$

$\lambda_{(280\ nm)}$ = 1900 $M^{-1}.cm^{-1}$

B.

$A = 0.0032*[C]$
$r^2 = 0.998$

$\lambda_{(400\ nm)}$ = 3200 $M^{-1}.cm^{-1}$

Figure S 18: Determination of molar extinction coefficient of maleimide crosslink at A. 280 nm and B. 400 nm

Proteolysis studies on BID peptides

Trypsin, modified sequencing grade from bovine pancreas (lyophilized powder, MW = 24 kDa, ≥ 4 units/mg protein) was purchased from Sigma-Aldrich and used without further purification. BID peptides (200 μM stock in PBS buffer pH 7.50, 5% DMSO) were treated with Trypsin (0.02 μM stock solutions in PBS buffer pH 7.50) in a 1:10000 enzyme/substrate ratio. The degradation was followed with LCMS (Aeris Peptide LC-Column XB-C18 2.6 μm, 100x2.1 mm, injection volume: 5 μL, acetonitrile/water (0.1% formic acid) 5-95% gradient).

The degradation pattern of each peptide (figure A) could be identified by mass spectrometry. The cleavage sites are marked by dotted lines and the subsequent fragment masses, shown in squares, are allocated to a number which is reported on the LCMS graph (graph B).

The LCMS traces (graph B) of each peptide incubated with Trypsin were recorded at the following successive times: 0, 5, 12, 21, 45 and 90 min.

From these data, the intensity of the mass corresponding to the entire peptide sequence (graph C) was integrated to extract kinetic values.

LCMS traces of RED-BID-Cys 1a in presence of Trypsin

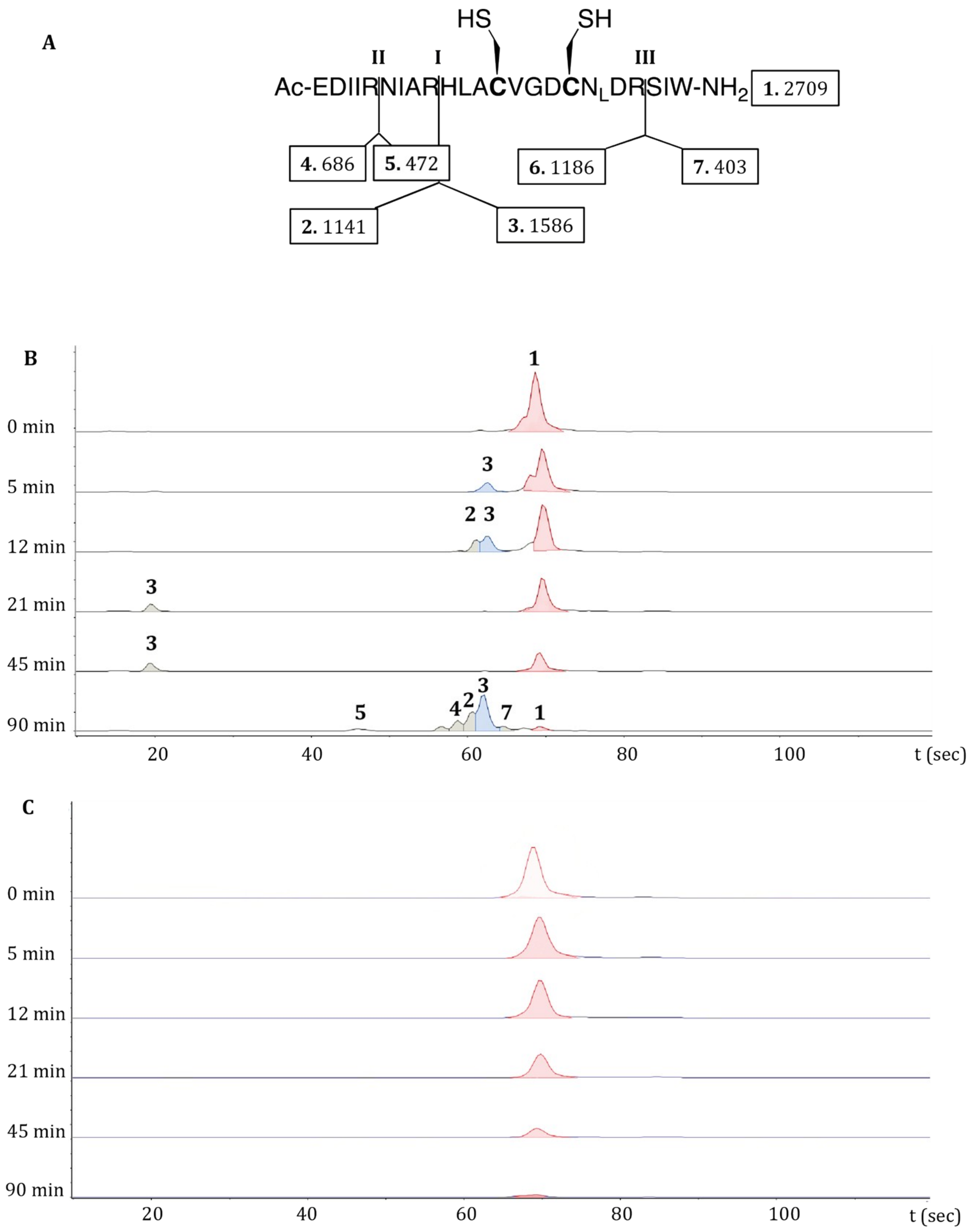

Figure S 19: A. Degradation pattern of 1a; B. MS traces; C. MS traces of the full-length peptide

LCMS traces of STA-BID-Cys 3a in presence of Trypsin

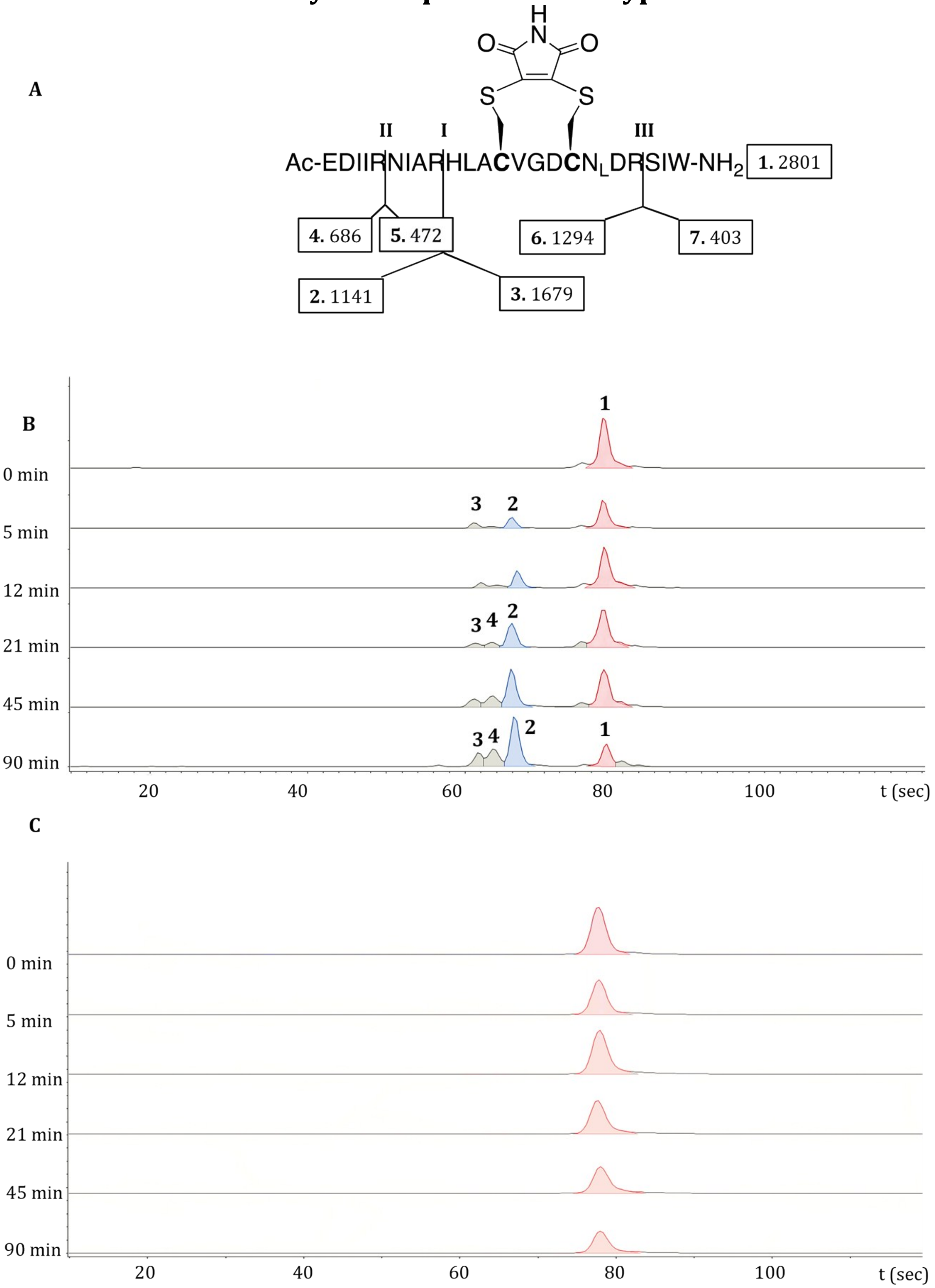

Figure S 20: A. Degradation pattern of 3a; B. MS traces; C. MS traces of the full-length peptide

LCMS traces of RED-BID-hCys 1b in presence of Trypsin

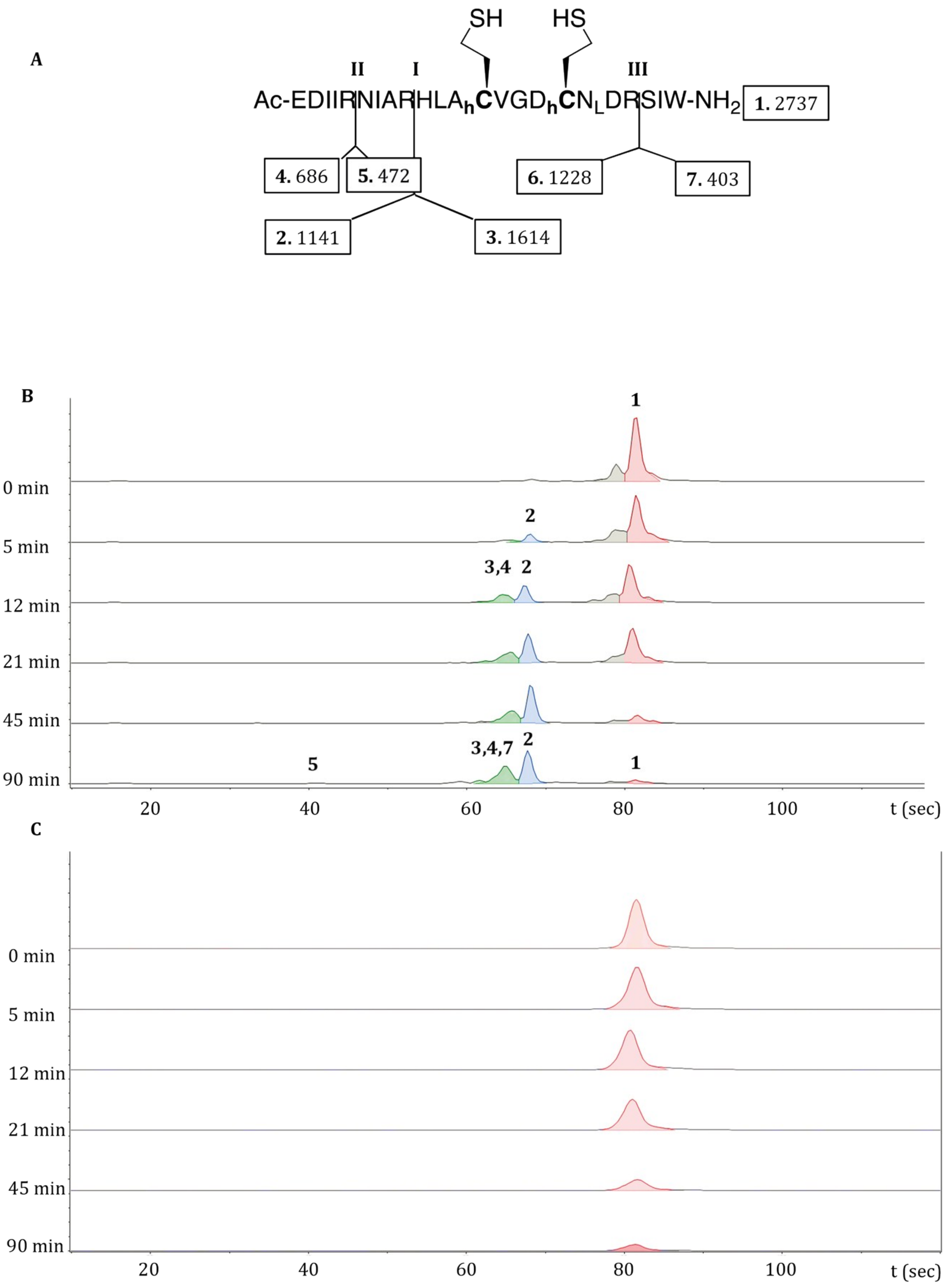

Figure S 21: A. Degradation pattern of 1b; B. MS traces; C. MS traces of the full-length peptide

LCMS traces of STA-BID-hCys 3b in presence of Trypsin

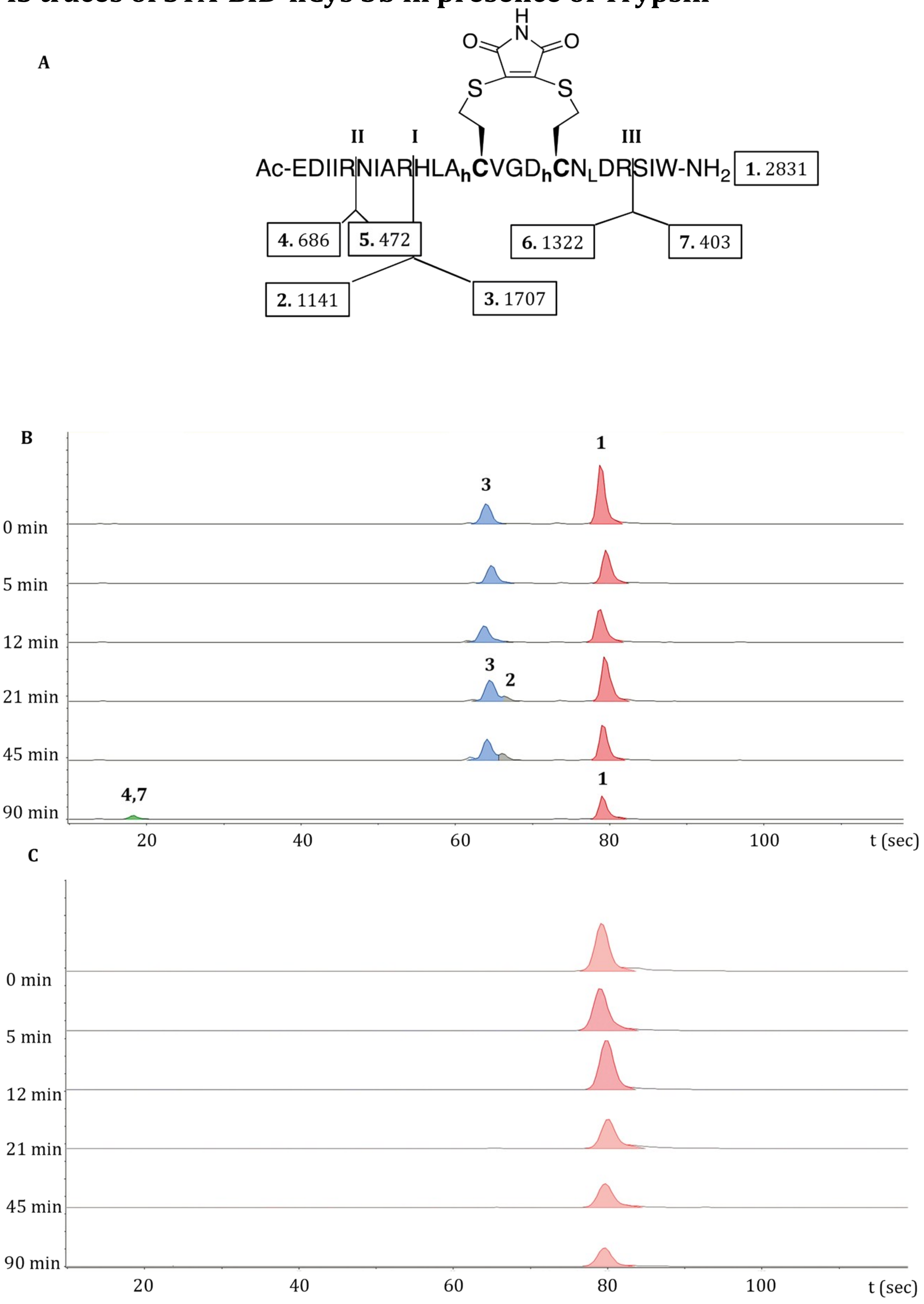

Figure S 22: A. Degradation pattern of 3b; B. MS traces; C. MS traces of the full-length peptide

Circular Dichroism

Samples were prepared in two different solvent systems (30% TFE:H_2O, 30% CH_3CN:water) to concentrations between 10 - 20 µM (Figure S 23). Considering the high number of hydrophobic residues in BID and S peptides, organic co-solvents were used to ensure good solubility and avoid aggregation at concentrations high enough for CD analyses. DMSO stocks of peptides were not used for CD due to its high absorbance below 230 nm.

The raw circular dichroism data obtained for the peptides was processed by the subtraction of the solvent signal and converted into a mean residue ellipticity:

$$[\theta]_{MRE} = \frac{\theta}{(10 * c * l)(R - 1)}$$

Where $[\theta]_{MRE}$ = molar ellipticity per residue, θ = circular dichroism at a given wavelength, c = molar concentration, l = path length in cm, R = number of residues in the peptide sequence.

Calculation of % helicity was performed by the following equation:[4]

$$\theta_H = \left(\theta_0 + \frac{d\theta}{dt}T\right) * (1 - \frac{x}{N_r})$$

Where: θ_H = Theoretical MRE for 100% helicity at 222 nm, θ_0 = Theoretical MRE for 100% helicity at 222 nm at 0 °C = –44000, (∂θ/∂T) = temperature dependence of infinite helix = +250, T = temperature in °C, x = 3, N_r = number of residues in the peptide.

In both solvent systems, it is noteworthy that STA-peptides are more helical than RED-peptides or WT-peptides; with the exception of constrained S-Cys-peptides. The use of TFE as co-solvent in water enhances the α-helicity of both series of peptides compared to the use of acetonitrile in water. The CD analyses show that STA-peptides can adopt a helical (and thus bioactive) conformation. The S peptides show a low helix propensity which is consistent with the observations of Hamachi[5] and hence TFE has a more dramatic effect.

BID peptides **S peptides**

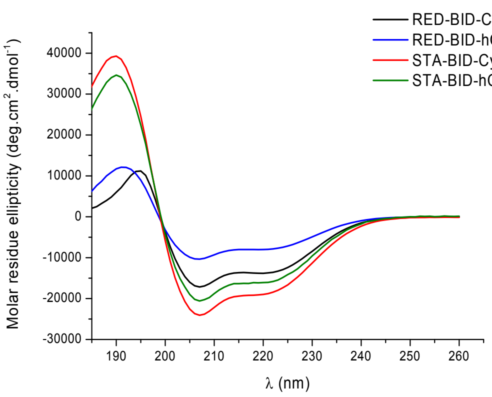

A.

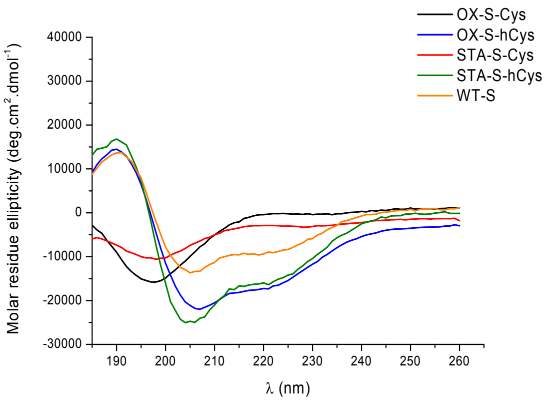

B.

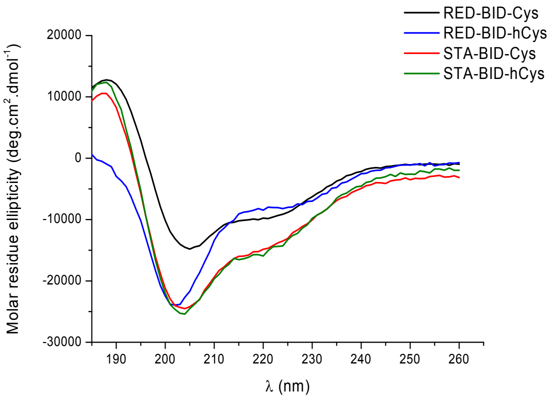

C.

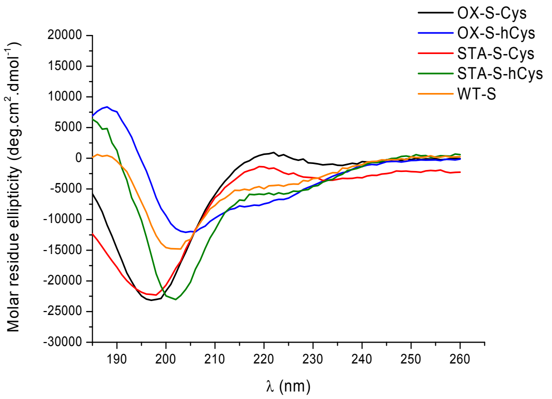

D.

Figure S 23: CD spectra A. and B. in 30% TFE:H_2O; C. and D. in 30% CH3CN:H_2O.

Table 1: Percentage of helicity in the three solvent systems

Peptides	30% TFE:H_2O	30% CH_3CN:H_2O
RED-BID-Cys – **1a**	40%	29%
RED-BID-hCys – **1b**	23%	24%
STA-BID-Cys – **3a**	55%	42%
STA-BID-hCys – **3b**	47%	43%
WT-S – **8**	30%	15%
OX-S-Cys – **2c**	0%	0%
OX-S-hCys – **2d**	56%	23%
STA-S-Cys – **3c**	9%	5%
STA-S-hCys – **3d**	52%	20%

Molecular Modelling

Molecular Modelling allowed us to study two different features:

- the influence of the maleimide cross-link on helicity was studied by modelling RED-BID-hCys **1b** as the non constrained peptide model and STA-BID-Cys **3a** as the stapled peptide model.
- the length of the constraint ring on helicity was studied by modelling STA-S-Cys **3c** and STA-S-hCys **3d** and WT-S **8** was used as a control.

Molecular Mechanics calculation

A hybrid MCMM conformational search was carried out on **1b**, **3a**, **8**, **3c** and **3d** in a water medium using Macromodel software package from Schrödinger and the MMFF force field with the following restraint: the dihedral angles of each amino acid were restricted to –58° for ϕ and to –47° for ψ.
The calculation was run to generate 1 000 conformer sets, of which the lowest energy conformer was retained for inspection. As anticipated, all lowest energy conformers adopted an α-helical conformation and showing a complete series of α-helical intramolecular hydrogen bonds (Figure S 24).

RED-BID-hCys **1b** STA-BID-Cys **3a** STA-S-Cys **3c** STA-S-hCys **3d**

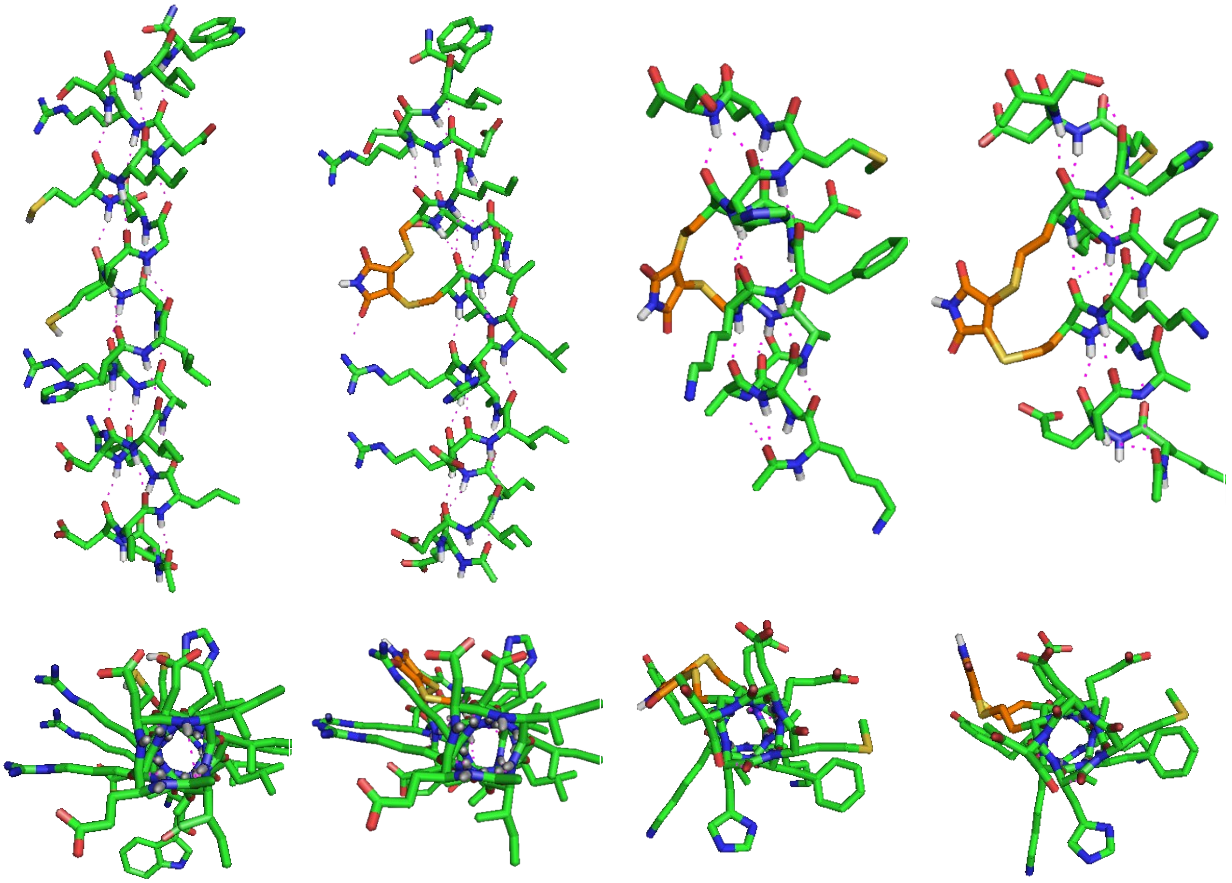

Figure S 24: Side and top views of the structures of the lowest energy helical conformers.

Molecular Dynamics simulation

The molecular dynamics simulation was conducted using the Desmond software package from Schrödinger. The lowest-energy conformer of **1b**, **3a**, **8**, **3c** and **3d** (generated by the MCMM calculation described above) was placed in a cubic cell, with the size adjusted to maintain a minimum distance of 10 Å to the cell boundary and soaked with an orthorhombic box of TIP3P using the System Builder module. The OPLS 2005 force field was used to model all peptide interactions. The system was equilibrated with the default protocol of Desmond then a NPT production simulation with all restraints removed was run at 300 K and 1 atm for 10 ns. Configurations were saved at 20 ps intervals.

The existence of hydrogen bonds was determined via a geometric criterion requiring a maximum donor (NH)-to-(O=C) acceptor distance of 2.5 Å and a maximum N–H–O=C angle of 30°.

Comparison between RED-BID-hCys 1b and STA-BID-Cys 3a

The intramolecular hydrogen bonds were monitored and displayed as a 2D graph (Figure S 25). The X axis corresponds to the simulation time. The Y axis reports the total number of intramolecular hydrogen bonds within the peptide.

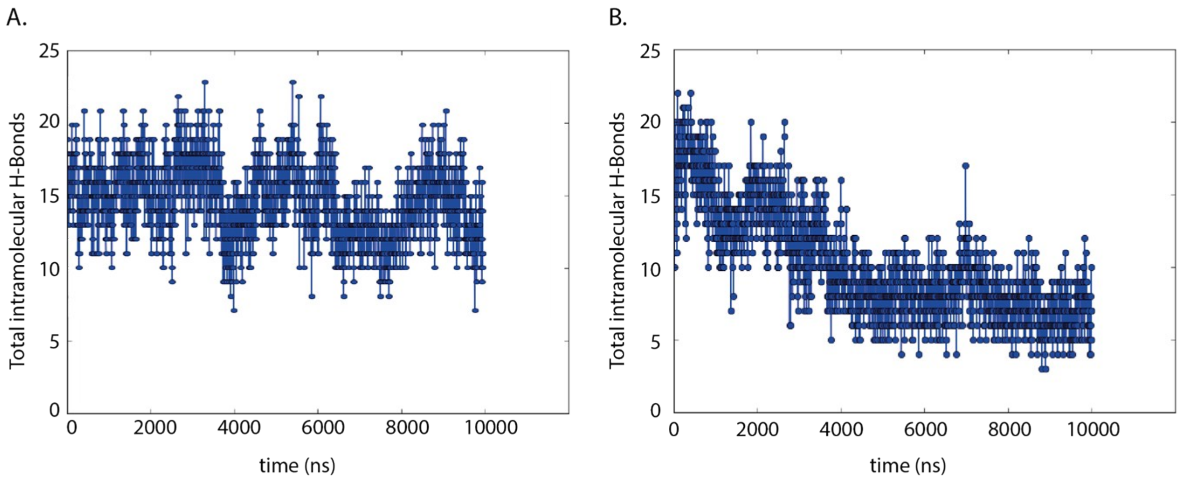

Figure S 25: Total number of hydrogen bonds of A. STA-BID-Cys and B. RED-BID-hCys monitored during the 10 ns simulation time

The MD simulations clearly show that the total number of intramolecular H-bonds is greater in STA-BID-hCys than in RED-BID-Cys, illustrating the increased stability of STA-BID-peptides.

The occurrence of helical hydrogen bonds was monitored and displayed as a 2D graph (Figure S 26). The X axis corresponds to the simulation time. The Y axis reports the presence, 1, or the absence, 0, of intramolecular hydrogen bonds within the peptide.

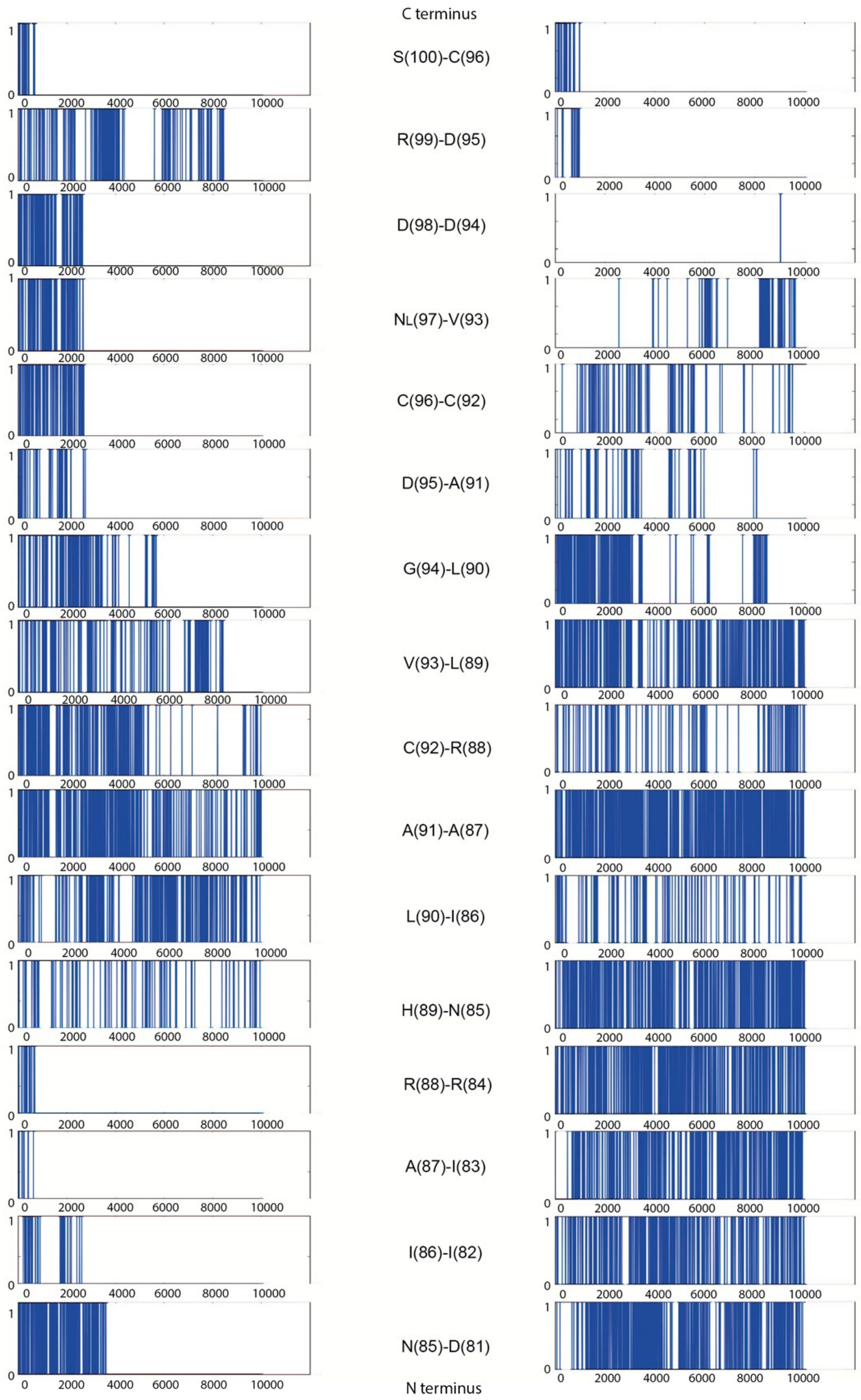

Figure S 26: Occurrence of each helical hydrogen bond of BID-peptides during monitored the 10 ns simulation time: RED-BID-hCys (left) and STA-BID-Cys (right).

The analysis of H-bonds showed that helical H-bonds are more prominent during the simulation in STA-BID-hCys than in RED-BID-Cys. The MD experiments confirm that the α-helical conformation of STA-BID peptides is more stable than RED-BID peptides, consistent with CD analyses.

Comparison between WT-S 8, STA-S-Cys 3c and STA-S-hCys 3d

The intramolecular hydrogen bonds were monitored and displayed as a 2D graph (Figure S 27). The X axis corresponds to the simulation time. The Y axis reports the total number of intramolecular hydrogen bonds within the peptide.

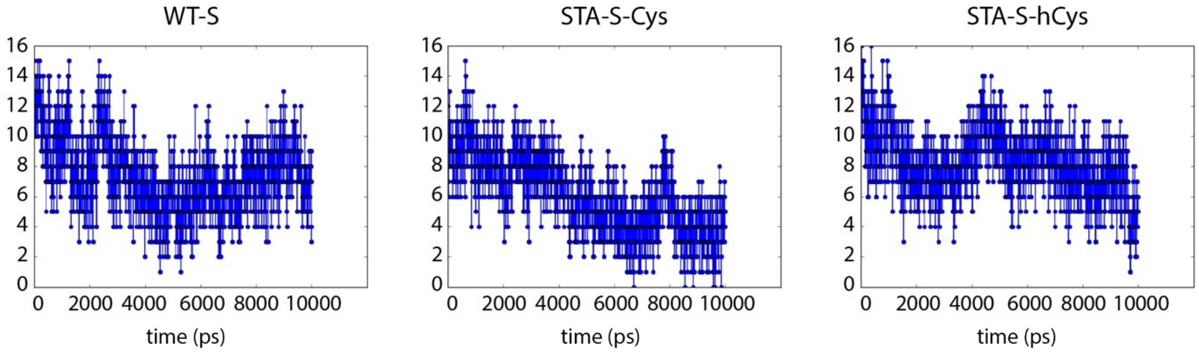

Figure S 27: Total number of hydrogen bonds of WT-S, STA-BID-Cys and STA-S-hCys monitored during the 10 ns simulation time

During the entire simulation, the total number of intramolecular H-bonds is greater in STA-S-hCys than in STA-S-Cys and in WT-S, illustrating the increased stability of STA-S-hCys, consistent with CD analyses.

The occurrence of helical hydrogen bonds was monitored and displayed as a 2D graph (Figure S 28). The X axis corresponds to the simulation time. The Y axis reports the presence, 1, or the absence, 0, of intramolecular hydrogen bonds within the peptide.

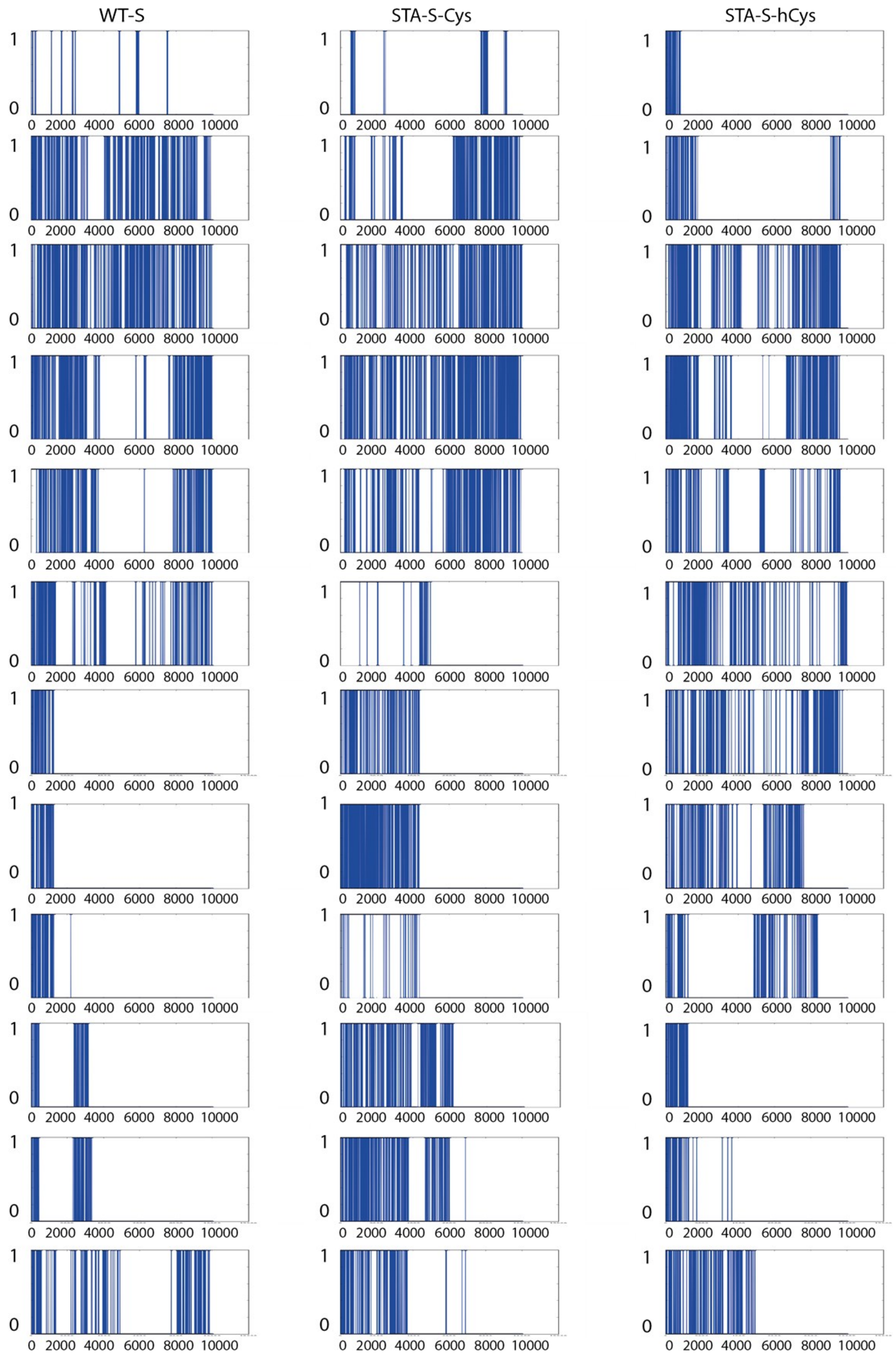

Figure S 28: Occurrence of each helical hydrogen bond of S-peptides during monitored the 10 ns simulation time: WT-S 8, STA-S-Cys 3c, STA-S-hCys 3d.

The analysis of H-bonds showed that helical H-bonds are more prominent during the simulation and along the entire sequence in STA-S-hCys than in WT-S and STA-S-Cys. The

helical H-bonds in STA-S-Cys considerably drop after 6 ns illustrating the instability of the α-helical conformation, consistent with the CD analyses.

Fluorescence Anisotropy

The fluorescein-labelled FITC-Ahx-NOXA B (75-92) was purchased from Severn Biotech Ltd, the labelled BODIPY-TMR-BAK (572-586) was purchased from ProteoGenix and the fluorescein-labelled FITC-Ahx-BID (80-102) was synthesised and purified as stated previously.[6]
Fluorescence anisotropy assays were performed in 384-well black plates (Greiner Bio-one). Each experiment was run in triplicate and fluorescence anisotropy was measured using a Perkin Elmer EnVisionTM 2103 MultiLabel plate reader. Fluorescein labelled peptides used an excitation and emission wavelength of 480 nm (30 nm bandwidth) and 535 nm (40 nm bandwidth) respectively (polarised dichroic mirror at 505 nm) whilst BODIPY labelled peptides used an excitation and emission wavelength of 531 nm (25 nm bandwidth) and 595 nm (60 nm bandwidth) respectively (polarised dichroic mirror at 555 nm).

Experiments were performed in different assay buffers according to the targeted protein and the tracer:

- 50 mM Tris buffer at pH 7.4, containing 150 mM NaCl and 0.61% of Triton X-100 for FITC-BID/Mcl-1.
- 50 mM phosphate buffer at pH 7.5, containing 200 mM NaCl and 0.02 $mg.mL^{-1}$ bovine serum albumin (BSA) for BODIPY-BAK/Bcl-x_L.
- 50 mM phosphate buffer at pH 7.5, containing 200 mM NaCl and 0.05% of tween for FITC-NOXA B/Mcl-1.

20 μL of assay buffer were first added to each well.

For each competition assay, 20 μL of a solution of α-helix mimetics (1 mM in 90:10 (v/v) assay buffer: DMSO) were added to the first column. The solution was well mixed and 20 μL were taken out and added to the next column and so on. This operation consisted on serial dilution of the peptides across the plate (24-points, 1/2 serial dilution). The tracer peptide and the targeted protein were added to each well to give a final concentration of 25 nM and 150 nM, respectively.

For each direct titration assay, 20 μL of a solution of the protein (60 μM in assay buffer) were added to the first column. The solution was well mixed and 20 μL were taken out and added to the next column and so on. This operation consisted on serial dilution of the protein across the plate (24-points, 1/2 serial dilution). The tracer peptide (225 nM stock solution in assay buffer) was then added to each well to give a final concentration of 75 nM of tracer peptide and 15 μM of the protein.

For control wells in both assays, the tracer peptide was replaced with an identical volume of assay buffer. The total volume in each well was 60 μL. Plates were read after 1 h of incubation at room temperature.

The data for both the P (perpendicular intensity) and S (parallel (same) intensity) channels, resulting from this measurement and corrected by subtracting the corresponding control

wells, were used to calculate the intensity and anisotropy for each well following Equations 1 and 2:

$$I = (2PG + S) \quad \text{(Equation 1)}$$

$$r = \frac{S - PG}{I} \quad \text{(Equation 2)}$$

Where I is the total intensity, G is an instrument factor which was set to 1 for all experiments and r is the anisotropy.

The average anisotropy (across three experimental replicates) and the standard deviation of these values were then calculated and fit to a sigmoidal logistic model (Equation 3) using OriginPro 9.0 which provided the IC50 and error values.

$$y = r_{max} + \frac{r_{min} - r_{max}}{1 + (\frac{x}{x_0})^p} \quad \text{(Equation 3)}$$

Competitive assay

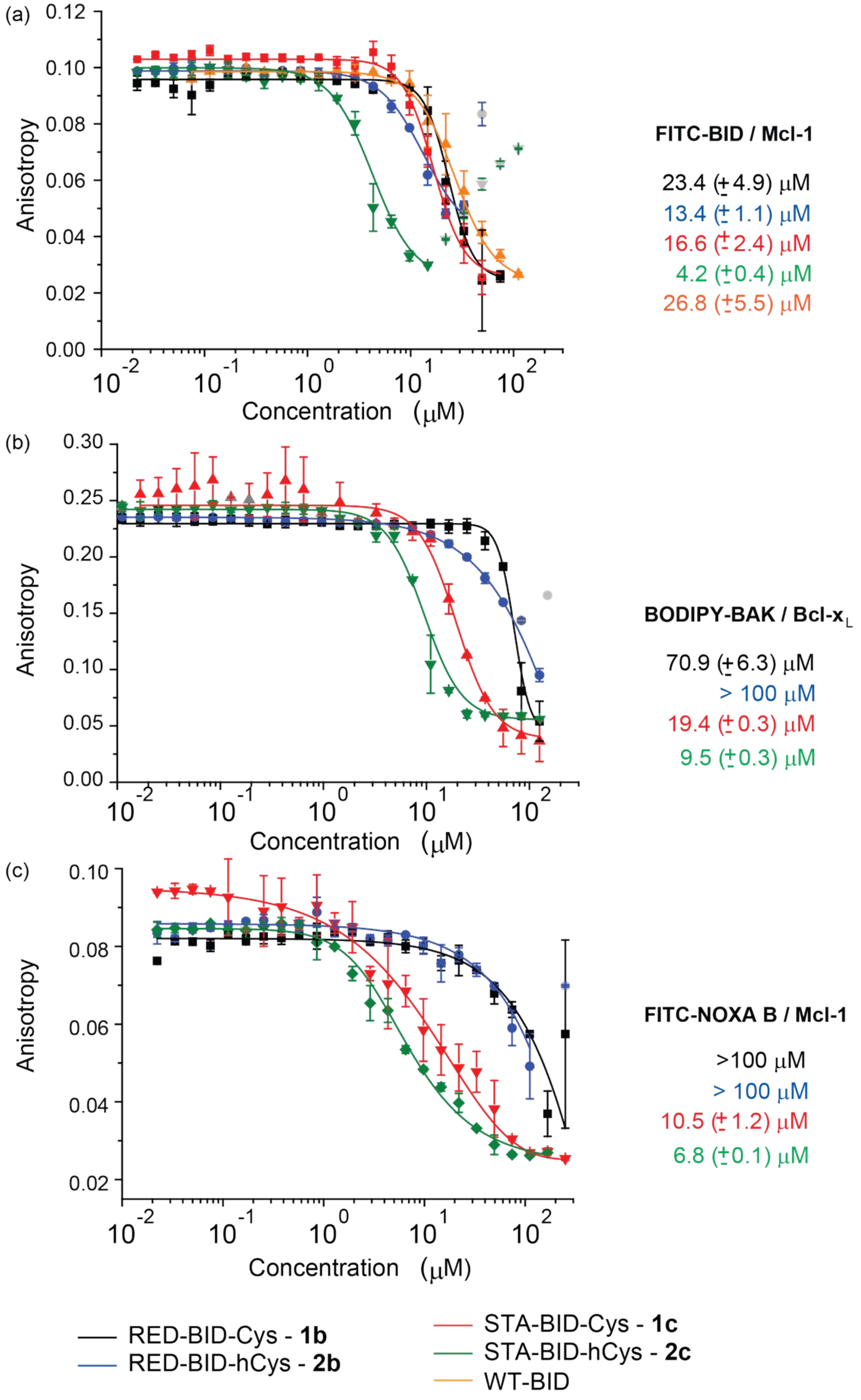

Figure S 29: Additional fluorescence anisotropy competition assay for the inhibition of A: FITC-BID/Mcl-1; B: BODIPY-BAK/Bcl-x$_L$; C: FITC-NOXA B/Mcl-1 (the grey points have been removed for fitting)

Direct binding assay

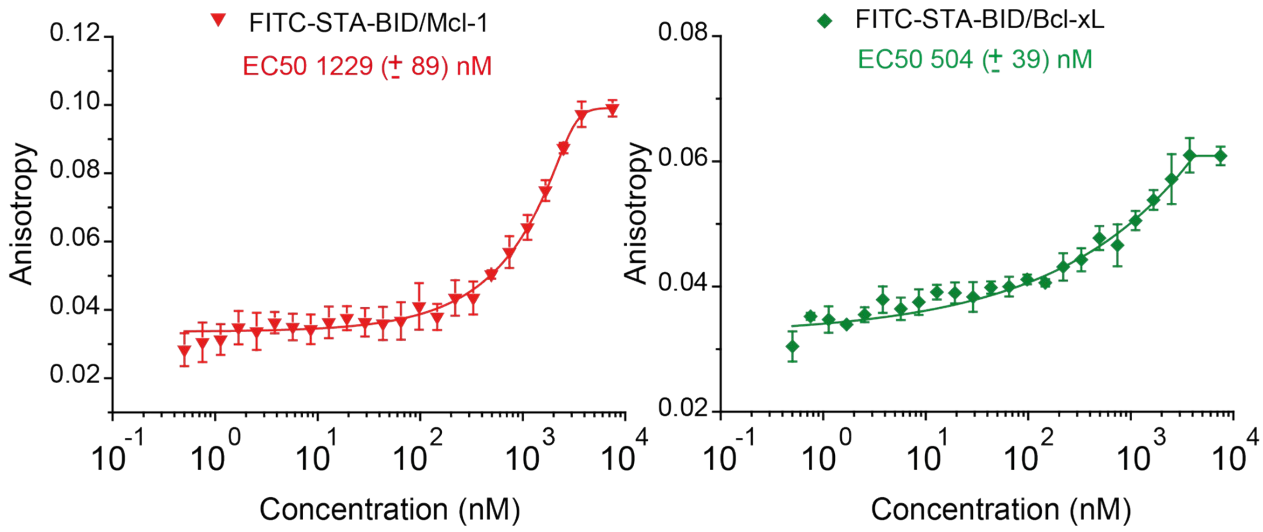

Figure S 30: Fluorescence anisotropy direct binding assay for interaction of BID peptides with Mcl-1 and Bcl-x_L

RNA degradation assay

Preparation of S protein

RNase A (10 mg) in Hepes (600 μL, 100 mM ; pH = 8.0) was digested by subtilisin (5 mg/mL, 10 μL) for 4 hrs at 0 °C. After which, the buffer was acidified with a solution of HCl (1 M) to reach pH = 3. The acidic solution was stirred for 1 h at 2 °C. The reaction was monitored by HPLC using the Jupiter μ Proteo 90 A analytical column using a gradient (0-60% over 30 min). After completion of the reaction, a solution of 20% trichloroacetic acid (20% v/v) was added to precipitate out the S protein. The sample was centrifuged and the precipitate collected and suspended in water. After dialysis in phosphate buffer (20 mM of sodium phosphate, pH = 5), S protein was purified from RNase A on a small SP Sepharose column using sodium phosphate (200 mM, pH = 6.6) and sodium acetate (20 mM) giving S protein (5.2 mg, 62%). HRMS (ESI): 11445.4, 11532.6 Da found, S protein 22-124 / S protein 21-124 calcd 114446.3 / 115333.3 Da.

Conditions of the assay

RNA degradation assays were performed in 96-well black plates (Greiner Bio-one). Each experiment was run in triplicate and the fluorescence was measured using a Perkin Elmer EnVisionTM 2103 MultiLabel plate reader, with an excitation and emission at 510 nm and at 600 nm.[7]

Before the experiment, RNA (1 mg/mL) was mixed with a solution of Ethidium Bromide (EtBr) (0.077 mM) in water for 30 min in a falcon tube covered in foil.

The buffer used in this enzymatic assay was Tris buffer (50 mM of Tris, 100 mM of NaCl, pH = 7.5). The adequate quantity of buffer was first added to each well to reach a total volume of 200 μL (150 μL for controls, 149 μL for 0.1 μM of S peptides, 148 μL for 0.2 μM of S peptides, 145 μL for 0.5 μM of S peptides, 140 μL for 1 μM of S peptides, 130 μL for 2 μM of S peptides). 20μL of S protein (0.5 μM stock solution in buffer) were added to each well. The adequate quantity of S peptides (0μL for controls, 1 μL for 0.1 μM, 2 μL for 0.2 μM, 5 μL for 0.5 μM, 10 μL for 1 μM, 20 μL for 2 μM) from a 20 μM stock solution in buffer was added to each well. 30μL of RNA were finally added to each well.
For positive control wells, the S protein was replaced with the RNase A (0.5 μM stock solution in buffer).
For negative control wells, the S peptides were replaced with an identical volume of assay buffer.

Plates were immediately read after the addition of EtBr-labelled RNA and after 60 min of incubation at room temperature.

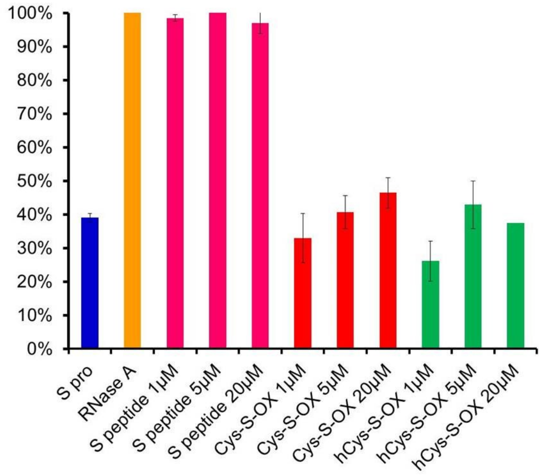

Figure S 31: RNA degradation assay showing the failure to restore S protein activity with oxodised peptides 2c and 2d. S protein was used as a negative control, and RNase A as a positive control. (50 mM Tris, 100 mM NaCl, pH 7.50).

Bibliography

[1] M. W. Jones, R. A. Strickland, F. F. Schumacher, S. Caddick, J. R. Baker, M. I. Gibson, D. M. Haddleton, *J. Am. Chem. Soc.* **2012**, *134*, 1847–1852.

[2] A. Barnard, K. Long, D. J. Yeo, J. A. Miles, V. Azzarito, G. M. Burslem, P. Prabhakaran, T. A. Edwards, A. J. Wilson, *Org. Biomol. Chem.* **2014**, *12*, 6794–6799.

[3] A. Barnard, K. Long, H. L. Martin, J. A. Miles, T. A. Edwards, D. C. Tomlinson, A. Macdonald, A. J. Wilson, *Angew. Chem. Int. Ed.* **2015**, *54*, 2960–2965.

[4] P. Luo, R. L. Baldwin, *Biochemistry (Mosc.)* **1997**, *36*, 8413–8421.

[5] I. Hamachi, Y. Yamada, T. Matsugi, S. Shinkai, *Chem. – Eur. J.* **1999**, *5*, 1503–1511.

[6] J. A. Miles, D. J. Yeo, P. Rowell, S. Rodriguez-Marin, C. M. Pask, S. L. Warriner, T. A. Edwards, A. J. Wilson, *Chem. Sci.* **2016**, DOI 10.1039/C5SC04048E.

[7] D. R. Tripathy, A. K. Dinda, S. Dasgupta, *Anal. Biochem.* **2013**, *437*, 126–129.
